# Causal effects of breastfeeding promotion on child health: understanding the role of nutrition

**DOI:** 10.1186/s41937-026-00153-0

**Published:** 2026-05-12

**Authors:** Anne Ardila Brenøe, Jenna Stearns, Richard M. Martin

**Affiliations:** 1 Department of Business Administration, University of Zurich, Plattenstrasse 14, 8032 Zurich, Switzerland; 2 Department of Economics, University of California, Davis, 1 Shields Avenue, Davis, CA 95616 USA; 3 Bristol Medical School, University of Bristol, Canynge Hall, 39 Whatley Road, Bristol, BS8 2PS UK

**Keywords:** Breastfeeding, Infant feeding, Child health, The Promotion of Breastfeeding Intervention Trial (PROBIT), I10, J13, J24

## Abstract

Using data from the only large-scale randomized controlled trial promoting prolonged exclusive breastfeeding, we study how the intervention affected child health and why. The intervention increased weight-for-age in infancy, with effects persisting through adolescence. We show that treated infants were breastfed more and received less water, juice, and other liquids, resulting in a more calorie-dense diet. A mediation analysis indicates that increased caloric intake explains a large share of the early weight gain, while reduced illness explains little. These findings suggest that, in this setting, the main benefits of breastfeeding promotion for physical growth came from improved nutrition. More broadly, the results highlight that the effects of breastfeeding promotion depend on the local alternatives to breast milk and may differ in settings where infant formula or other more nutritious substitutes are the main alternative.

## Introduction

The potential positive association between exclusive breastfeeding and beneficial child outcomes has extensive public health and economic consequences across the world.^1^ Initiatives to limit or regulate the marketing of infant formula, enact workplace breastfeeding policies, and promote paid leave to encourage breastfeeding are based on the idea that breastfeeding causally improves child outcomes. But despite a large medical and epidemiological literature on breastfeeding (e.g., (Kramer & Kakuma, 2012; Victora et al., 2016)), the causal evidence of the effects on child outcomes is scarce (Fitzsimons & Vera-Hernández, 2022). Arguably, the best evidence comes from the Promotion of Breastfeeding Intervention Trial (PROBIT) in Belarus—the only large-scale randomized controlled trial (RCT) on breastfeeding to date. This hospital-level intervention was based on the WHO/UNICEF Baby-Friendly Hospital Initiative and substantially increased the duration and exclusivity of breastfeeding (Kramer et al., 2001). In this RCT, the overall benefits of the promotion of breastfeeding were fewer than those found in observational settings, which are prone to issues of bias and reverse causation (Kramer, 2010).

A key limitation of the current causal literature is the lack of evidence on mechanisms. Any benefits of breastfeeding on child health and development could be driven by improved infant nutrition or caloric intake, the unique elements of breast milk (such as antibodies) that are not found in high-quality infant formula, or increased social stimulation through the physical act of breastfeeding. Understanding which of these mechanisms are most relevant is crucial for designing optimal infant feeding policies—whether it is to promote and support breastfeeding itself, enable breast pumping at work, regulate the composition of infant formula, or to promote programs to more broadly foster early childhood stimulation. In this paper, we provide novel evidence suggesting that improved nutrition is a key driver of the benefits of breastfeeding.

We use data from the PROBIT RCT to estimate the causal effects of promoting breastfeeding duration and exclusivity on child health and provide novel insight into the role of nutrition as a plausible mechanism. We first show that the intervention significantly and persistently increased weight-for-age, but find little evidence of robust and persistent effects on several other dimensions of child health. As our main contribution, we next provide novel evidence on how the breastfeeding promotion intervention affected infant feeding patterns and nutrition and how these changes mediated the effect on infant weight gain. We find that infants exposed to the breastfeeding intervention were breastfed more and consumed less water, juice, and other liquids throughout their first year of life. This resulted in a more calorie-dense and calorie-rich diet compared to infants in the control group.

These effects on infant feeding patterns are important for interpreting the mediating mechanisms of breastfeeding on child health. We employ a mediation analysis that shows that the increased caloric intake explains a large proportion of the effect on weight gain in early infancy. This suggests that improved nutrition during key periods of growth has contemporaneous and lasting effects on weight gain. The results also indicate that, at least in this setting where water and juice are common substitutes for breast milk, the primary benefit of breastfeeding on children’s outcomes is improved nutrition. In contrast, we do not find any evidence that reduced incidence of illness can explain the effects on weight gain, ruling out the unique elements of breast milk not found in infant formula as an important mechanism of weight gain. Furthermore, there is no strong evidence of heterogeneity in the effects of the breastfeeding promotion intervention on child health or feeding patterns with respect to gender or socioeconomic status and, consistent with previous research, we do not find supportive evidence of effects of the intervention on socioemotional skills at age 6 (Kramer et al., 2008a) or cognitive development at age 16 (Yang et al., 2018).^2^ These results suggest that improved nutrition in infancy is the main mechanism driving the effects on weight gain.

Our findings have important policy implications and underscore the need for continued research in this area. The results highlight the importance of understanding the local alternatives to breastfeeding when evaluating the potential benefits of breastfeeding interventions. Because mothers in Belarus did not use breast milk as a perfect substitute for infant formula, but instead replaced breastfeeding with less-nutritious alternatives, the results on weight gain may represent an upper bound of the health benefits of breastfeeding in other settings. It is not clear that breastfeeding interventions would affect weight gain in populations where infant formula is already widely used as the primary alternative to breast milk, as is the case in the USA (Grummer-Strawn et al., 2008). Although more work is needed, evidence on the causal effects of breastfeeding on early childhood health in high-income countries is extremely limited (Baker & Milligan, 2008; Del Bono and Rabe 2012; Fitzsimons & Vera-Hernández, 2022).

We contribute to the literature in several important ways. Our key contribution is to bring new insights into the mechanisms of breastfeeding. Previous PROBIT papers have not reported the effect of the intervention on other infant feeding outcomes than breastfeeding exclusivity and duration. However, to interpret the effects of the intervention and for thinking about the generalizability of the results, it is important to understand what the alternatives to breast milk were and how the intervention affected infant feeding patterns. We further contribute to the existing literature analyzing the effect of the PROBIT intervention on child development by advancing the empirical framework. Overall, our findings on child outcomes are consistent with previous findings (Kramer et al., 2001, 2002, 2008a; Martin et al., 2017; Yang et al., 2018).

Our results also contribute to a literature on the effects of nutritional supplementation among infants and toddlers on contemporaneous weight gain and later cognitive development in developing countries (Maluccio et al., 2009; Schroeder et al., 1995; Walker et al., 1991, 2005). In contrast with these studies, it is worth noting that our findings are from a setting in which infants were not generally underweight or particularly unhealthy even in absence of the breastfeeding intervention. By providing the first causal evidence linking the positive effects of breastfeeding to improved nutrition among healthy infants, we highlight the importance of understanding mechanisms by which breastfeeding can improve child outcomes when designing cost-effective policies that support optimal infant feeding practices.

## The PROBIT study

The PROBIT study was a cluster RCT in Belarus based on the WHO/UNICEF Baby-Friendly Hospital Initiative’s “10 Steps to successful breastfeeding.” As the only large-scale breastfeeding RCT conducted among healthy full-term infants, it was designed to identify the causal effects of breastfeeding promotion among mothers who had expressed a prenatal intention to breastfeed on breastfeeding duration and infant health (Kramer et al., 2001). Randomization occurred at the hospital level (the cluster), and treatment hospitals were given extensive training in methods to promote and prolong breastfeeding, maintain lactation, and resolve common problems.

We use data on 16,774 children born between June 1996 and December 1997 in 30 maternity hospitals.^3^ Eligible infants were born weighing at least 2500 grams and at a gestational age of 37 weeks or greater. The hospitals were geographically dispersed across Belarus and were matched in pairs stratified on region, degree of urbanization, the annual number of deliveries, and the pre-intervention breastfeeding initiation rate. Treatment status within each hospital pair was assigned randomly. More details about the PROBIT design are available in Kramer et al. (2000).

Across all hospitals, the postpartum stay after a routine vaginal delivery was 6–7 days (Kramer et al., 2001). Treatment hospitals received an intervention promoting breastfeeding, while the control hospitals continued the standard practices in effect at the time of randomization. The conventional practices at control sites included routine separation of mother and child, delayed onset of breastfeeding, scheduled (versus on demand) feedings, routine use of water, formula, and other liquids in newborn diets, and recommendation of early introduction of solid foods (Patel et al., 2013). In contrast, mothers at intervention hospitals received help initiating breastfeeding within 30 min of a normal birth, infants were supposed to remain with their mothers 24 h a day during the postpartum hospital stay, and newborns were fed only breast milk on demand unless medically indicated. All pregnant women at intervention hospitals were also informed about the benefits and management of breastfeeding. The intervention required hospitals assigned to the active group to have a written breastfeeding policy that all staff had the skills to implement. The head obstetrician and pediatrician from each treatment hospital received an 18 h lactation management training course, and then, trial participants organized and implemented further training programs for midwives, nurses, physicians, and pediatricians working in the postpartum ward and pediatricians working in the associated polyclinic. The full implementation of the intervention required at least 12 months. Further details about the experimental context are provided in Appendix A.2.

As previously documented by Kramer et al. (2001), the breastfeeding promotion intervention substantially changed mothers’ breastfeeding behavior. Mothers who gave birth at treated hospitals were considerably more likely to breastfeed exclusively for up to six months compared to mothers at control hospitals.^4^ For example, treated mothers were two and six times more likely to exclusively breastfeed infants at ages one and three months, respectively, compared to mothers in the control group who had exclusive breastfeeding rates of only 27 percent at one month and seven percent at three months. The intervention also increased breastfeeding duration, with treated mothers significantly more likely to breastfeed for at least 12 months.

During the late 1990s, Belarus resembled Western developed countries in terms of basic health services and sanitation. The country had high rates of adult literacy and immunization, and low rates of infant and child mortality. Maternity and postpartum infant care practices were comparable to those in North America and Western Europe 20 years earlier. At the time of the study, locally made infant formula was readily available but expensive. Exclusive use of infant formula costs nearly 20 percent of the average monthly salary, compared to about 2.5 percent of the median monthly income in the USA. However, other social supports for new mothers were relatively generous. For instance, in contrast with the USA, mothers in Belarus had three years of maternity leave (often obligatory), possibly making it relatively easier for mothers to breastfeed.

## Empirical strategy

We analyze the intent to treat (ITT) effects of the PROBIT breastfeeding promotion intervention by estimating the following specification:

1 $$\begin{aligned} Y_{iph}=\gamma _0+\gamma _1\mathrm{Treatment}_h+Z_i'\delta +\theta _p+\varepsilon _{iph}, \end{aligned}$$

where $Y_{iph}$ is the outcome of interest for individual *i* at a specific age, born at hospital pair *p*, and hospital *h*. The variable *Treatment* is an indicator for whether the hospital received the breastfeeding intervention. We control for a vector of individual baseline characteristics, *Z* (birth weight in grams (squared); maternal and paternal age (squared); indicators for gender, cesarean section, gestational age at birth in weeks, maternal smoking and alcohol use during pregnancy, parents’ marital and cohabitation status, number of siblings, maternal and paternal educational attainment, and quarter-by-year of birth). Appendix Table A1 shows descriptive statistics at baseline. As expected, given the RCT design, mothers who gave birth at treated and control hospitals are very similar. Finally, $\theta _p$ are hospital pair fixed effects, which we include to account for the stratified design (Duflo et al., 2007).

We allow for the errors, $\varepsilon _{iph}$, to be correlated at the hospital level. Due to the small number of clusters, we use the wild cluster bootstrap (WCB) to estimate p-values (Cameron et al., 2008) and conduct 999 replications. This is in contrast with the clustering methods used in previous published PROBIT papers and may explain differences in the precision of the respective effect estimates.^5^ Finally, because we estimate effects for a number of related outcomes, all results are corrected for multiple hypothesis testing using the method developed in Benjamini et al. (2006). Known as the *krieger* method, this is a “step-up” method of multiple hypothesis testing that is less data intensive than methods designed for use in very large samples, and controls for the false discovery rate (FDR) rather than the family-wise error rate.

## Data and outcome variables

Participants were followed six times throughout their first year of life at regular health checkups (at 1, 2, 3, 6, 9, and 12 months), and again at ages 6.5, 11.5, and 16.^6^ In each wave, a pediatrician conducted physical growth measurements. For the analysis of child health, we focus on weight-for-age, which we construct as a standardized age-specific z-score (Vidmar et al., 2004). Children in the control group had healthy weights during infancy and childhood (Appendix Table A3). Using a standardized weight-for-age measure yields an easy interpretation of the results on weight gain in terms of standard deviations. Particularly in early life, it also is highly correlated with recent changes in nutrition and health status.^7^ This makes it a well-suited measure for studying the relationship between breastfeeding, nutrition, and physical growth.

Our infant feeding data are based on maternal reports of feedings during the 24 h before each of the six infant checkups. We use information on the frequency and volume of feedings of breast milk, infant formula, cow’s milk, water, juices or other liquids, and solid food (including cereals). Less than one percent of breast milk feedings is expressed milk and less than 0.1 percent is donor milk, with the rest being breastfeedings at the breast. Thus, we refer to breast milk and breastfeeding interchangeably. Based on the reported frequency and volume of feedings from each category, we also construct measures of caloric intake. We assume breast milk, formula, and cow’s milk contain 65 kcal per 100 ml, while juices and other liquids contain an average of 30 kcal per 100 ml.^8^ For breast milk and solid foods, a measure of volume per feeding is not available. We follow Lupton et al. (2002) to estimate breast milk caloric intake and volume.^9^ As for every type of self-reported measure, we cannot rule out potential reporting bias in the feeding data. However, mothers did not have any particular incentive to misreport.^10^

## Results

### Child health

**Fig. 1:**
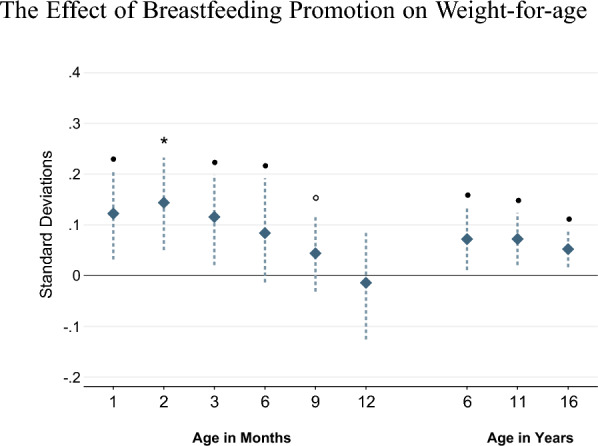
Effect of Breastfeeding Promotion on Weight-for-age. *Note:* Each estimate comes from a separate regression as specified in Eq. (1); the dashed lines show the 95 percent confidence interval based on wild cluster bootstrapped (WCB) standard errors clustered at the hospital level. Multiple hypothesis testing using the *krieger* method is performed on all estimates within the graph. Significance levels after testing for multiple hypothesis are indicated as follows: $\circ p<0.10$, $\bullet p<0.05$, *$p<0.01$

Figure 1 shows the effects of the breastfeeding promotion intervention on weight-for-age throughout childhood. Overall, we find a statistically significant and persistent effect of the intervention on weight-for-age. Infants exposed to the intervention were about 0.10 standard deviations heavier during the first six months of life compared to those in the control group. Interestingly, the effects on weight re-emerged later in childhood and persisted throughout adolescence. At age 16, children in the treatment group had a 0.04 standard deviation higher weight-for-age. These findings are consistent with those reported by Kramer et al. (2002) and Martin et al. (2017) from the PROBIT trial . Children were not statistically more likely to be either overweight or underweight at any of the considered ages (Appendix Figure A7), suggesting that the effect on weight-for-age was driven by weights within the healthy range.

Considering other dimensions of child health, we overall confirm previous findings from the PROBIT study (Appendix A.5). In line with Kramer et al. (2001), the breastfeeding intervention decreased the likelihood of infant illness, although our effects are imprecisely estimated (Appendix Figure A8). We also do not find meaningful effects on a physical health index or persistent effects on height-for-age (Appendix Figure A7); these findings are consistent with those by Kramer et al. (2002, 2007) and Martin et al. (2013, 2017). Thus, within the domain of child health, the only robust and persistent effect of the breastfeeding intervention is on weight gain.^11^

### Infant feeding patterns

**Fig. 2:**
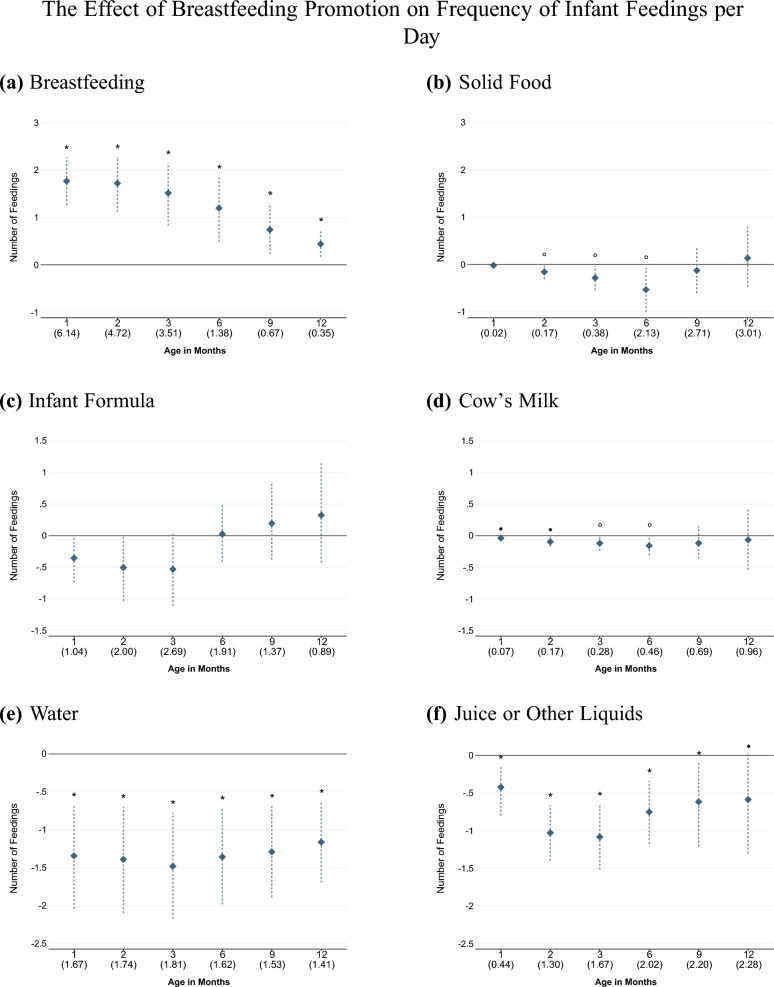
Effect of Breastfeeding Promotion on Frequency of Infant Feedings per Day.*Note:* Each estimate comes from a separate regression as specified in Eq. (1); the dashed lines show the 95 percent confidence interval based on wild cluster bootstrapped (WCB) standard errors clustered at the hospital level. Multiple hypothesis testing using the *krieger* method is performed on all estimates within the same graph. Significance levels after testing for multiple hypothesis are indicated as follows: $\circ p<0.10$, $\bullet p<0.05$, *$p<0.01$. The numbers reported in parenthesis on the horizontal axis indicate the control mean of the respective outcome variable. Each outcome measures the number of feedings of that particular liquid or food that the infant received during the previous 24 h.

**Fig. 3:**
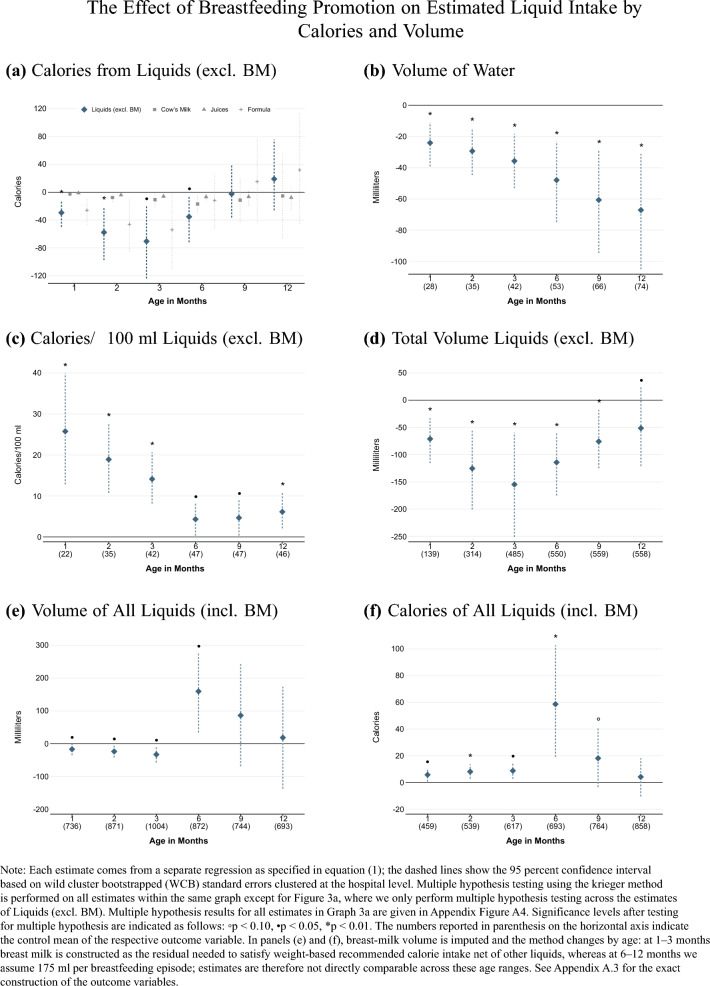
Effect of Breastfeeding Promotion on Estimated Liquid Intake by Calories and Volume

Studying child health and development, we only find robust statistically significant effects of the breastfeeding promotion intervention on weight-for-age. This effect persisted until at least age 16, but appears to be driven by early weight gain during months also associated with higher rates of exclusive breastfeeding. What can explain these weight gain effects?

To examine the nutrition mechanism through which breastfeeding promotion affected weight, we turn to detailed data on infant feeding patterns. Figure 2 shows the number of feedings per day over the first 12 months of life by nutrition type (breast milk, solid food, infant formula, cow’s milk, water, and juice or other liquids). As expected, given earlier work on breastfeeding rates, infants exposed to the treatment were breastfed about 1.7 more times per day during the first three months of life, and 0.8 more times per day between 6 and 12 months. This corresponds to approximately 428 more breastfeedings during the first year, an increase of 50 percent relative to the control group.

While the frequency of infant formula feedings seemed to decrease during the first three months of life, the biggest substitutes for breastfeeding in terms of the number of feedings were water and juice, which are much less nutritionally rich. Infants in the treated group received about 1.4 and 0.7 fewer feedings per day of water and juice or other liquids, respectively, compared to those in the control group, and this persisted throughout the full first year of life. There was also a small reduction in the frequency of receiving cow’s milk and solid food during the first six months.^12^ Thus, we observe a stronger compliance to the recommendation of first introducing solid food at age six months in the treatment group.

Next, we estimate differences in the volume and caloric intake from liquids other than breast milk (Graphs 3a –3d of Figure 3). The intervention reduced the daily caloric intake from liquids excluding breast milk by about 50 kcal during the first six months. Although water and juice were the biggest substitutes for breast milk in terms of feeding frequency, the decrease in the caloric intake from infant formula among treated infants was ten times larger than that from juices. We also see a substantial reduction in the volume of water consumed throughout the first year of life. Together, these findings imply an increase in the density of calories treated infants received. Treated infants received around 20 kcal more per 100 ml liquids (excluding breast milk) during the first three months in particular, compared to control infants. Consistent with these results, treated infants received a smaller total volume of liquids other than breast milk on the order of 50–150 ml per day compared to infants in the control group.

Finally, we estimate the total volume of and caloric intake from liquids including breast milk. Because we do not observe the quantity of breast milk consumed (only the frequency of breastfeeding), this analysis is naturally associated with more measurement error, as we must estimate the volume per feeding. At ages one to three months, for breastfed infants we impute breast milk intake using the weight-based recommended daily calorie intake formula and treating breast milk as the residual after subtracting calories from other reported liquids. Thus, Graphs 3e and 3f partly mechanically reflect substitution between breast milk and other liquids (and variation in recommended intake due to infant weight), rather than a fully observed measure of realized total energy intake. From six months onwards, when solid foods become quantitatively important and we lack nutritional information on solids, we instead assume a fixed volume of 175 ml per breastfeeding episode; this value is conservative relative to commonly cited per-feeding milk volumes at these ages (roughly 180–240 ml) (American Academy of Pediatrics, 2019).

When including breast milk in the volume of all liquids, the estimated effect is very small for ages one to three months and becomes positive at age six months (Graph 3e ).^13^ Graph 3f shows the estimated total caloric intake from all liquids including breast milk. Treated infants received around eight calories more per day from liquids than control infants at ages one to three months; the larger estimates at later ages should be interpreted with caution given the change in breast milk imputation and the increasing (unobserved) role of solid foods. At the same time, because our assumed 175 ml per breastfeeding episode is conservative relative to commonly cited per-feeding milk volumes at these ages, the estimated differences in calories from liquids at 6–12 months are, if anything, likely to be attenuated. The increased caloric intake from liquids represents almost a two percent increase in calories per day compared to the recommended nutritional guidelines for infants. It is thus evident that the breastfeeding promotion intervention increased both the nutritional density and the total amount of calories received from liquids. One relevant data limitation is that we do not know which type of solid food infants received or how much they ate. Because there is little evidence of meaningful differences in solid food feedings between the treatment and control group, we interpret the results for liquids to imply that treated infants received more calories during their first year of life.

Interestingly, early breastfeeding appears to have a lasting effect on feeding patterns. Even after mothers exposed to the breastfeeding intervention stop exclusively breastfeeding, they are less likely to feed less-nutritious liquids, such as water or juice, than are control mothers, and are more likely to delay the introduction of solid food until the child reaches six months of age. Treated mothers are also actually somewhat more likely to use infant formula after they stopped exclusively breastfeeding, which is much more nutritionally rich and similar to breast milk than other liquids. These patterns may suggestively indicate that breastfeeding leads to a longer-term change in nutrition that could potentially explain the persistent effects on weight gain through adolescence. In support of this idea and in line with Skugarevsky et al. (2014), we also find a robust decline in an index measuring problematic eating attitudes among treated children at age 11 (Appendix Figure A5).

### The role of nutrition in explaining infant weight gain

Can the increase in calorie consumption due to the breastfeeding promotion intervention explain the effects on weight gain? Following Gelbach (2016), we decompose the treatment effect on weight-for-age into experimentally induced changes in caloric intake and changes in other (unmeasured) factors. We limit this mediation analysis to ages one to three months because calories received from solid foods are minimal at these ages; thus, we have good estimates for the total calorie intake of all infants during this period.

We calculate the mediated effect, ME, of the experimentally induced increase in the total caloric intake on weight-for-age as:

2 $$\begin{aligned} \mathrm{ME} = \underbrace{\frac{\partial Y_{iph}}{\partial \mathrm{Calories}_i}}_\text {A} \underbrace{\frac{\partial \mathrm{Calories}_i}{\partial \mathrm{Treatment}_h}}_\text {B}, \end{aligned}$$

following the notation from Eq. (1). The term B of Eq. (2) represents the causal effect of the intervention on the calorie intake. The term A is the parameter $\phi$ from the following equation:

3 $$\begin{aligned} Y_{iph} = \alpha _0 + \alpha _1 \mathrm{Treatmemt}_h + \phi \mathrm{Calories}_i + Z_i'\delta ^{'} + \theta _p^{'} +\varepsilon _{iph}^{'}. \end{aligned}$$

Under the assumption that Eq. (3) does not omit any variables that both affect the mediator (*Calories*) and the outcome, we can interpret the estimates from the mediation analysis as causal. Although we cannot think of any obvious omitted variables, we can naturally not rule out this possibility. We then calculate the mediated effect as the share of the total treatment effect of the intervention on weight-for-age. In the mediation analysis, we include as mediator the total cumulative calorie intake from all liquids including breast milk through the age at the weight measurement.

**Fig. 4:**
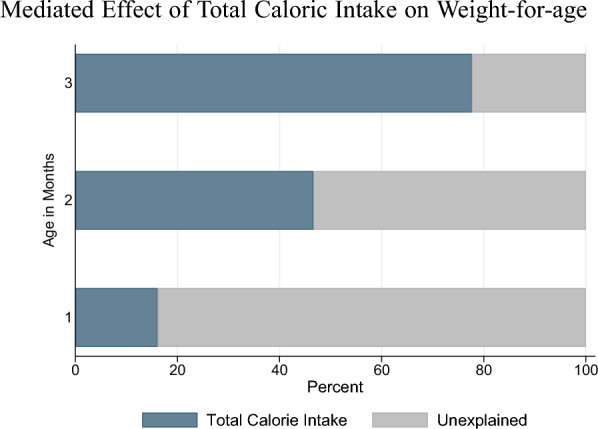
Mediated Effect of Total Caloric Intake on Weight-for-age. *Note:* This figure shows the results of the mediation analysis, decomposing the overall treatment effect on weight-for-age at age one, two, and three months into the experimentally induced changes in the total cumulated caloric intake from all liquids including breast milk and changes in other (unmeasured) factors. At one, two, and three months, respectively, the p-values of the estimate of the mediator are 0.068, $<0.001$, and $<0.001$. For the estimation, we use the Gelbach (2016) b1x2 Stata package

Figure 4 presents the results from the mediation analysis. We estimate that a remarkable 78 percent of the effect of the breastfeeding intervention on weight-for-age is mediated by the experimentally induced increase in the total calorie intake at age three months. The effect of the intervention on caloric intake becomes increasingly important over the first few months of life, with 46 percent of the effect explained by increased calorie consumption at age two months and a third of this at age one month.

This analysis suggests that improved nutrition is the predominant mechanism for the effects of the breastfeeding intervention on weight gain. However, other channels might potentially also be relevant. In addition to feeding practices, the BFHI package promoted immediate mother–infant skin-to-skin contact after delivery and discouraged routine early separation. The clinical evidence for healthy term newborns points to consistent benefits for breastfeeding initiation (and modest improvements in thermal/glucose stabilization), with limited evidence that skin-to-skin contact has sustained independent effects on infant growth beyond breastfeeding (Moore et al., 2025). Relatedly, the increase in breastfeeding also implies an increased exposure of infants to maternal antibodies and thereby a potential improvement in infants’ immune system response (De Moraes-Pinto et al., 2021). Although we do not find statistically significant effects on the infant illness index, we cannot rule out medium-sized reductions. Appendix Table A2 includes the illness index as an additional mediator. Only a negligible part of the treatment effect of the intervention on weight-for-age is mediated through this index. The results are similar when including each of the components separately. In particular, gastroenteritis in early infancy does not explain any of the effect on weight gain which is worth highlighting as gastroenteritis typically involves diarrhea or vomiting and thereby likely reduces caloric intake.

In addition to the increased exposure to biological components in breast milk, the intervention may also have reduced the exposure to non-sterile food or feeding equipment, which might have improved infant health beyond what our illness index captures. In Appendix Table A2, we therefore consider as mediators the number of the different types of feedings. None of the non-breast milk feeding types have any mediating power, but the number of breastfeedings does mediate a substantial fraction of the effect of the breastfeeding intervention on weight gain. Thus, there is no support of this potential channel, which is also in line with the generally good hygienic setting.

When considering the important factors generally affecting body weight among older children and adults, physical exercise is key in addition to nutrition. However, it seems unlikely that physical activity would be an important mechanism in our setting, as young infants typically sleep 13–16 h per day (Iglowstein et al., 2003) and spend most of the remaining time eating. Finally, we also find no evidence of significant socioemotional or cognitive effects (Appendix Figure A9) that could be driven by the physical act of breastfeeding.

In conclusion, while it is likely that all of these potential mechanisms may matter to some degree, nutrition appears to be by far the most important factor through which breastfeeding impacts children’s physical growth in this setting.

## Conclusion

WHO’s recommendation of six months of exclusive breastfeeding has extensive public health and economic consequences. However, this global recommendation is based on limited causal evidence from specific policy settings. Little is known about the external validity of the effects of breastfeeding on child health and development, or the specific mechanisms that might drive these effects. Answering these questions is important from a policy perspective in order to issue efficient and cost-effective recommendations to support optimal infant feeding practices across the world.

In this paper, we study the causal effects of a breastfeeding promotion intervention on infant feeding patterns and childhood health using data from the PROBIT study—the only large-scale RCT on breastfeeding to date. Consistent with prior work, we confirm that infants exposed to the breastfeeding intervention had a significantly and consistently higher weight-for-age from infancy through at least age 16. More importantly, we provide novel insights on the effects of the intervention on the nutritional composition of the diet that infants received. Infants exposed to the breastfeeding intervention were breastfed more and received less water, juice, and other liquids throughout their first year of life. Although the infant feeding data are self-reported and potentially measured with error (as is typically the case for this type of data, with the Health and Demographic Surveys being a prominent example), these findings suggest that the intervention resulted in a more calorie-dense and calorie-rich diet.

In explaining the mechanism driving the effects of breastfeeding on child health, our analysis suggests that nutrition is the most important channel. While the increased calorie consumption of breastfed infants can explain most of the effect on weight gain in the first three months of life, reductions in illness explain less than 0.2 percent. This highlights the importance of changes in nutrition as the primary channel through which breastfeeding increased child weight gain in Belarus. This finding is relevant for similar settings characterized by low-nutrition alternatives to breast milk and is important for designing cost-effective policies that support optimal infant feeding practices. But it also stresses the importance of the specific policy environment in thinking about the generalizability of the results. We caution that the benefits of breastfeeding for child health and resulting policy implications may be different in settings where higher-nutrition alternatives to breast milk are more common.

## Additional file

Supplementary file 1 (pdf 6212 KB)

## Data Availability

The data used in this study are from the Promotion of Breastfeeding Intervention Trial (PROBIT), which are available under restricted access. Researchers can apply for access through the University of Bristol; more information is available here: https:// www.bristol.ac.uk/population-health-sciences/projects/probit/contact/. In accordance with the data availability policy, we will provide all results and do-files at a sufficient level of detail to reproduce the final results and will fully cooperate with any researcher seeking to conduct a replication of our results.

## Footnotes

1 The World Health Organization (WHO) recommends that mothers worldwide should exclusively breastfeed infants during the child’s first six months of life, and that children should continue to be breastfed in addition to receiving other nutritious foods until at least two years of age (WHO, 2011). Exclusive breastfeeding is defined as feeding no other foods or liquids, including water.

2 We report these results in Appendices A.5 and A.6.

3 Previous studies included 31 hospitals; however, we drop one unmatched hospital in our analysis. There is no evidence of differential attrition from the study by treatment status (Appendix Figure A1), and the intervention had no effect on infant mortality (Kramer et al., 2001). Additionally, trial staff estimated that only 1–2 percent of eligible women declined participation in the study.

4 Appendix Figure A2 shows the effects on exclusive and any breastfeeding.

5 Previous published PROBIT papers have used mixed effects linear models and mixed logistic regression models.

6 Appendix A.3 provides more details regarding the data and construction of the variables.

7 The results are qualitatively similar if we use weight or standardize the weight within the sample instead of weight-for-age (Appendix Figure A3).

8 Calories from juices and other liquids assume two-thirds comes from apple juice (45 kcal per 100 ml) and one-third from black tea (0 kcal per 100 ml).

9 For breastfed children at one to three months, we estimate breast milk intake based on the recommended daily calorie intake formula $\text {Calorie Intake} = 89 \times \text {Weight in Kg} + 75$, subtract their calorie intake from all other liquids, and calculate the volume. For breastfed children at 6 to 12 months, we assume that each breast milk feeding contains 175 ml (Whitehead et al., 1982). At these ages, we cannot use the daily calorie intake formula, as solid food makes up an increasing share of calorie intake and we do not have nutritional information about solid food.

10 We also note that we added the infant feeding analysis as a secondary analysis to our original analysis plan to investigate the channels of our results on child growth (for more details, see Appendix A.4).

11 We do not consider measures of eczema or asthma in our analysis. However, we note that Flohr et al. (2018) find a substantial reduction in flexural eczema on skin examination at age 16 by 54 percent, based on a rate in the control group of 0.7 percent.

12 We do not have information on the caloric intake of solid foods. However, we note that it is quite limited at ages one to three months, as infants this young are usually not able to digest or swallow non-liquids.

13 We note that the spike at age six months likely reflects delayed introduction of solid foods and the change in our breast milk imputation from six months onward; we therefore do not claim that this represents a net effect on total calorie intake at this age.
