# Supplementary material for: Causal effects of breastfeeding promotion on child health: understanding the role of nutrition
Source: Swiss J Econ Stat. 2026 May 12;162(1):8. doi: 10.1186/s41937-026-00153-0 (PMC13167875; doi:10.1186/s41937-026-00153-0)
Supplement: Supplementary file 1 — (pdf 6212 KB) [file 41937_2026_153_MOESM1_ESM.pdf]

Online Appendix

Causal Effects of Breastfeeding Promotion on Child Health: Understanding the Role of Nutrition

Anne Ardila Brenøe
Jenna Stearns
Richard M. Martin

March 30, 2026

A Online Appendix

A.1 Supplementary Figures and Tables

Figure A1
Attrition by Treatment Status

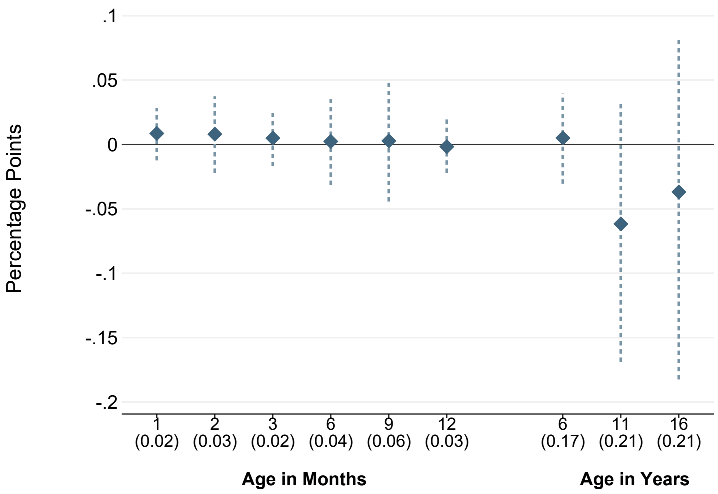

Note: Each estimate comes from a separate regression as specified in equation (1); the dashed lines show the 95 percent confidence interval based on wild cluster bootstrapped (WCB) standard errors clustered at the hospital level. Multiple hypothesis testing using the *krieger* method is performed on all estimates within the same graph. Significance levels after testing for multiple hypothesis are indicated as follows: $\circ p < 0.10$, $\bullet p < 0.05$, $*p < 0.01$. The numbers reported in parenthesis on the horizontal axis indicate the share of missing observations in the control arm at each time point. The number of non-missing observations for the treatment (control) arm for each wave (baseline, 1 month, 2 months, 3 months, 6 months, 9, months, 12 months, 6 years, 11 years, and 16 year) are 8,596 (8,178), 8,416 (8,078), 8,282 (7,985), 8,496 (8,123), 8,304 (7,916), 8,162 (7,771), 8,308 (7,921), 6,943 (6,788), 7,247 (6,472), 7,063 (6,491).

Figure A2
The Effect of Breastfeeding Promotion on Breastfeeding Exclusivity and Duration

(a) Exclusive Breastfeeding

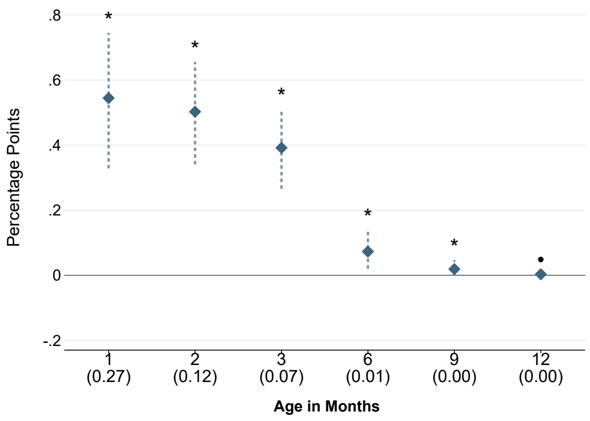

(b) Any Breastfeeding

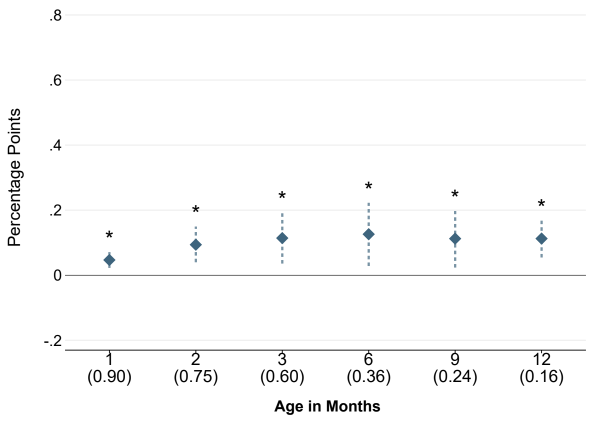

Note: Each estimate comes from a separate regression as specified in equation (1); the dashed lines show the 95 percent confidence interval based on wild cluster bootstrapped (WCB) standard errors clustered at the hospital level. Multiple hypothesis testing using the *krieger* method is performed on all estimates within the same graph. Significance levels after testing for multiple hypothesis are indicated as follows: $\circ p < 0.10$, $\bullet p < 0.05$, $^* p < 0.01$. The numbers reported in parenthesis on the horizontal axis indicate the control mean of the respective outcome variable.

Figure A3
The Effect of Breastfeeding Promotion on Weight

(a) Weight in Kilograms

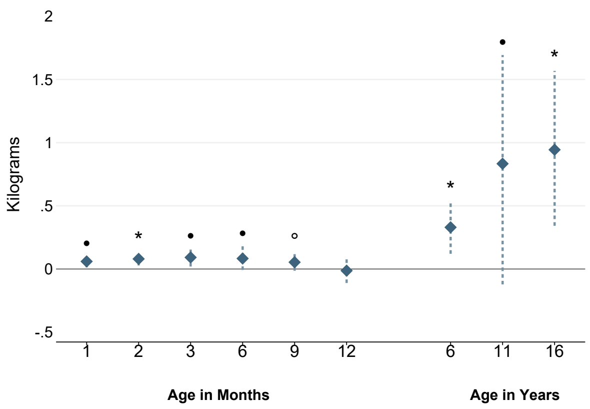

(b) Standardized Weight

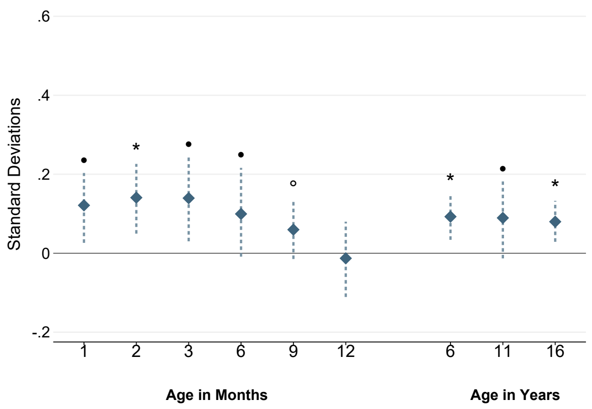

Note: The outcome in panel (b) is standardized with a mean of zero and standard deviation of one for the control group. Each estimate comes from a separate regression as specified in equation (1); the dashed lines show the 95 percent confidence interval based on wild cluster bootstrapped (WCB) standard errors clustered at the hospital level. Multiple hypothesis testing using the *krieger* method is performed on all estimates within the same graph. Significance levels after testing for multiple hypothesis are indicated as follows: $\circ p < 0.10$, $\bullet p < 0.05$, $^* p < 0.01$. The numbers reported in parenthesis on the horizontal axis indicate the control mean of the respective outcome variable.

Figure A4

The Effect of Breastfeeding on Estimated Infant Liquid Calorie Intake

(a) Calories from Liquids (excl. BM)

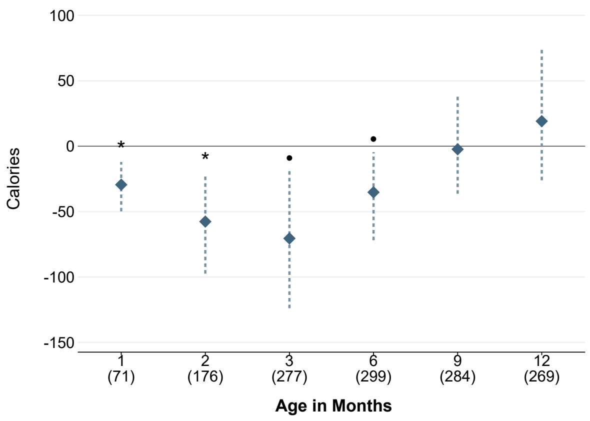

(b) Calories from Cow's Milk

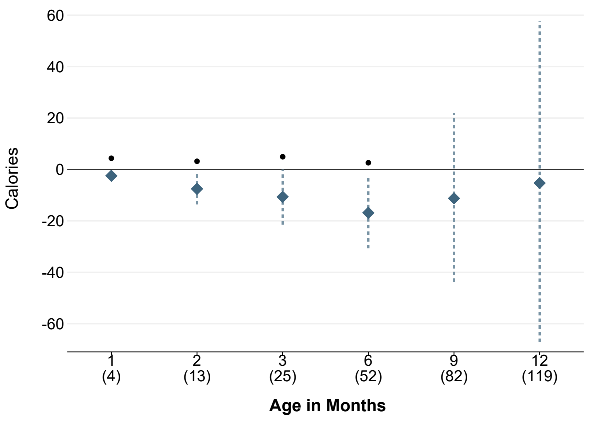

(c) Calories from Formula

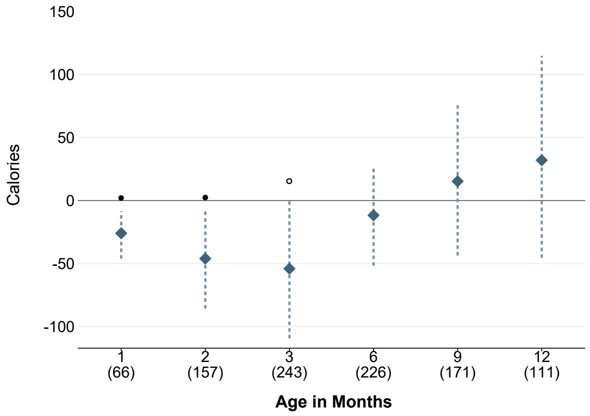

(d) Calories from Juices

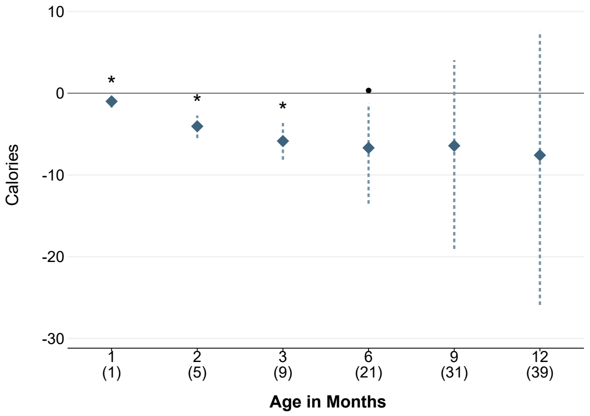

Note: Each estimate comes from a separate regression as specified in equation (1); the dashed lines show the 95 percent confidence interval based on wild cluster bootstrapped (WCB) standard errors clustered at the hospital level. Multiple hypothesis testing using the *krieger* method is performed on all estimates within the same graph. Significance levels after testing for multiple hypothesis are indicated as follows: $\circ p < 0.10$, $\bullet p < 0.05$, $*p < 0.01$. The numbers reported in parenthesis on the horizontal axis indicate the control mean of the respective outcome variable.

Figure A5
Treatment Effect on Children's Problematic Eating Attitudes

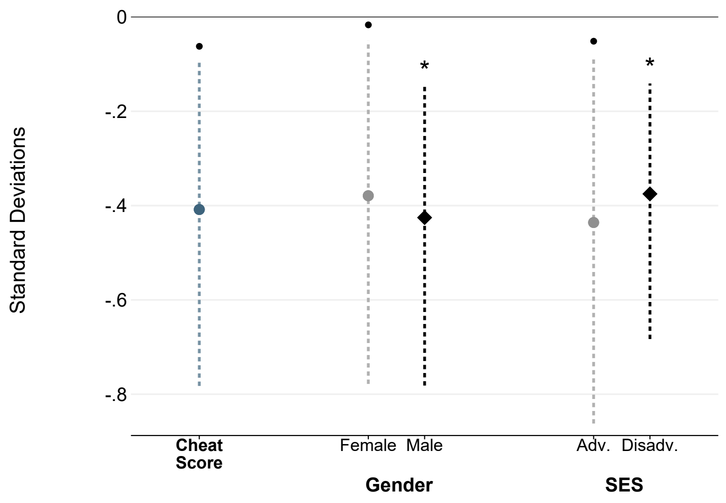

Note: The outcome is the Children's Eating Attitude Test (ChEAT), standardized with a mean of zero and standard deviation of one for the control group. A higher value indicates more problematic eating attitudes. For more details on the ChEAT measure, see Skugarevsky et al. (2014). The estimate most to the left comes from a separate regression as specified in equation (1), while the pairs of estimates come from regressions as specified in equation (4); the dashed lines show the 95 percent confidence interval based on wild cluster bootstrapped (WCB) standard errors clustered at the hospital level. Multiple hypothesis testing using the *krieger* method is performed on all estimates within the same graph. Significance levels after testing for multiple hypothesis are indicated as follows: ∘$p < 0.10$, •$p < 0.05$, *$p < 0.01$.

Table A1
Descriptive Statistics and Balancing Tests

	Control Mean (1)	Standard Deviation (2)	Difference (3)	P-value (4)
**Pregnancy and infant characteristics**				
Male	0.52	0.50	-0.00	0.85
Birth weight (g)	3437.91	420.98	3.21	0.78
Birth length (cm)	52.02	2.18	-0.21	0.49
Head circumference at birth (cm)	34.79	1.64	0.39	0.27
Delivery complications	0.08	0.28	-0.00	0.87
Caesarean section	0.10	0.31	0.02	0.17
Gestational age at birth (weeks)	39.33	0.98	0.16	0.20
Firstborn	0.56	0.50	0.04	0.11
Smoking during pregnancy	0.02	0.13	0.01	0.48
Alcohol during pregnancy	0.02	0.13	0.03	0.17
**Household characteristics**				
Married at birth	0.91	0.29	-0.04	0.00
Absent father	0.04	0.19	-0.01	0.52
Mother's Age (years)	24.44	4.91	-0.08	0.65
Father's Age (years)	27.34	5.15	-0.04	0.79
Number of other children in HH	0.58	0.86	-0.05	0.28
Previous exclusive breastfeeding $\geq$ 3 months	0.25	0.43	-0.00	0.96
Socio-economic disadvantage	0.36	0.48	-0.10	0.05
**Mother's education**				
University degree	0.13	0.34	0.01	0.59
Adv. secondary or partial university	0.53	0.50	-0.06	0.12
Secondary degree	0.30	0.46	0.04	0.36
Incomplete secondary	0.03	0.17	0.01	0.05
**Father's education**				
University degree	0.13	0.34	0.01	0.52
Adv. secondary or partial university	0.50	0.50	-0.10	0.08
Secondary degree	0.32	0.47	0.09	0.14
Incomplete secondary	0.06	0.23	0.00	0.97
**Mother's occupation**				
Agriculture or industry	0.32	0.47	0.03	0.65
Services	0.45	0.50	-0.05	0.33
Housewife	0.13	0.34	-0.00	0.74
Unemployed	0.07	0.26	0.01	0.36
Student	0.03	0.17	0.01	0.06
**Father's occupation**				
Agriculture or industry	0.50	0.50	0.05	0.46
Services	0.31	0.46	-0.06	0.37
Unemployed	0.14	0.35	-0.01	0.44
Student	0.01	0.10	0.01	0.00
**Observations**	Total: 16774; Treatment: 8596; Control: 8178			

Note: The table shows descriptive statistics at baseline. Columns (1) and (2) report the mean and standard deviation of each background variable for the control group. Column (3) shows the difference between the treatment and control groups, when accounting for hospital pair fixed effects. Of the 34 variables, six are statistically different at the 10 percent level, suggesting that families in the treatment and control groups are relatively comparable within hospital pairs. Due to imbalance in some of the characteristics, we include the rich vector of individual controls as explained in Section 3.

Table A2

Mediation Analysis: Additional Specifications

		1 Month				2 Month				3 Month		
	(1)	(2)	(3)	(4)	(5)	(6)	(7)	(8)	(9)	(10)	(11)	(12)
Total Calorie Intake	0.020	0.020	0.020		0.071	0.071	0.071		0.094	0.095	0.095	
	[0.068]	[0.069]	[0.069]		[0.000]	[0.000]	[0.000]		[0.000]	[0.000]	[0.000]	
Infant Health Index		0.002				0.002				0.005		
		[0.059]				[0.110]				[0.031]		
Any Rash			0.000				0.000				0.001	
			[0.386]				[0.308]				[0.179]	
Any Gastroentiritis			0.000				-0.001				0.000	
			[0.770]				[0.290]				[0.670]	
Any Respiratory			0.000				-0.000				0.000	
			[0.822]				[0.662]				[0.366]	
Any Other Illness			0.000				-0.000				-0.000	
			[0.773]				[0.896]				[0.738]	
Any Hospitalization			-0.000				-0.000				-0.000	
			[0.702]				[0.778]				[0.793]	
Freq. of Breast Milk				0.031				0.050				0.038
				[0.004]				[0.001]				[0.039]
Freq. of Formula				0.001				0.001				0.002
and Cow's Milk				[0.104]				[0.191]				[0.243]
Freq. of Juices and				-0.006				0.003				0.006
Other Liquids				[0.451]				[0.876]				[0.840]
Freq. of Water				0.022				0.024				0.009
				[0.030]				[0.166]				[0.542]
Est. eff. of interv. on WAZ at X months		0.122				0.152				0.121		
Mediated effect of	0.020	0.022	0.020	0.048	0.071	0.072	0.069	0.078	0.094	0.099	0.096	0.055
intervention	[0.068]	[0.031]	[0.053]	[0.010]	[0.000]	[0.000]	[0.000]	[0.007]	[0.000]	[0.000]	[0.000]	[0.085]
Mediated effect of interv. in percent	16.1	18.3	16.4	39.3	46.6	47.8	45.8	51.7	77.7	81.6	78.7	44.9
Est. eff. of interv.	0.103	0.100	0.102	0.074	0.081	0.079	0.082	0.073	0.027	0.022	0.026	0.067
on WAZ at X months with mediators	[0.000]	[0.000]	[0.000]	[0.007]	[0.000]	[0.000]	[0.000]	[0.013]	[0.236]	[0.333]	[0.263]	[0.043]

Note: This table reports the mediated effects based on a Gelbach (2016) decomposition of the intervention, using her b1x2 Stata package. The sample size is 10,152 for all specifications. We include the controls as specified in equation (1). The mediated effect of the intervention in percent is calculated using the ratio of the mediated effect of the intervention to the estimated effect of the intervention. The p-values generated by b1x2 packaged are reported below the estimates.

A.2 Experimental Content

The intervention was modeled on the Baby-Friendly Hospital Initiative (BFHI) developed by WHO and UNICEF and was a promotion and support of increased breastfeeding duration and exclusivity. The core aspects of the BFHI prescribe that the hospital should have a written breastfeeding policy that all staff should have the skills necessary to implement, mothers should be helped to initiate breastfeeding within half an hour after a normal birth, and unless medically indicated, newborn babies should have breast milk only. The head obstetrician and head pediatrician from each of the experimental maternity hospitals and polyclinics received the 18 hour BFHI lactation management training course organized by WHO. The aim of this course was to help hospitals transform their maternity facilities into baby-friendly institutions that implement the "Ten Steps to Successful Breastfeeding" and to assist them in implementing lasting policy changes.

The "Ten Steps to Successful Breastfeeding" are the following:

1. the hospital should have a written breastfeeding policy,
2. all staff should be trained in the skills necessary to implement the policy,
3. all pregnant women should be informed about the benefits and management of breastfeeding,
4. mothers should be helped to initiate breastfeeding within half an hour after a normal birth,
5. health workers should know how to assist in starting breastfeeding and how to maintain lactation during temporary separations,
6. unless medically indicated, newborn babies should have breast milk only,
7. babies should remain with their mothers 24 hours a day,
8. breastfeeding on demand should be encouraged,
9. pacifiers should not be given, and
10. the establishment of breastfeeding support groups should be fostered, and mothers should be referred to them on discharge (Kramer et al., 2000).

After the 18 hour course, the trial participants organized and implemented training programs for midwives, nurses, physicians, and pediatricians working in their postpartum ward and polyclinic, respectively. The full implementation of the intervention required at least 12 months.

A.3 Outcome Measures

This section provides additional details on the data and construction of the outcome measures in the main text as well as child outcomes shown in Appendix A.5. The PROBIT study consists of four waves conducted in infancy and at ages 6.5, 11.5, and 16 years. The first wave includes baseline data and data from six routine health checkups when the children were approximately 1, 2, 3, 6, 9, and 12 months.

Age at Measurement in Wave 1 Previous papers have considered the outcomes for each subwave during infancy regardless of infant age at the visit. In contrast, we define the outcomes by actual infant age and consider the ages in months as follows: 1 (< 1.5 months), 2 (1.5–2.5), 3 (2.5-4.5), 6 (4.5-7.5), 9 (7.5-10.5), and 12 (10.5–14). The subwaves generally correspond closely to the actual age; however, we reclassify 0.8 percent of the observations.

Summary Indices For the construction of the summary indices in the appendix, we reverse the signs of the components when necessary, so that all components in the indices indicate more favorable outcomes (except for the infant illness index; see below). Following Anderson (2008), we weight the standardized components by the covariance matrix and standardize the index so that the control group has a mean of zero and a standard deviation of one for each domain and age at survey. Following Kling, Liebman and Katz (2007), if an individual has a valid response to at least one component of a particular index, we impute any missing values for other component measures of that index with the mean of individuals from the same hospital, maternal education (low/high), and birth order (firstborn/non-firstborn). In addition to the indices, we also show the results for the individual components; these we do not impute. From an economic perspective, identifying significant effects on an index among related outcomes more strongly signals robust differences that may indicate important changes in health or development. It also is another way to limit identification of false positives when evaluating many related outcomes.

Physical Growth Index During all waves (including each of the subwaves during infancy), a pediatrician conducted anthropometric measurements. We consider the measures of height and weight and standardize these to age-specific z-scores based on the 2000 growth charts for the United States from the Centers for Disease Control and Prevention (Vidmar et al., 2004). We then construct a physical growth index for each wave which includes weight-for-age, height-for-age, body mass index (BMI)-for-age, and indicators for being underweight and overweight. We reverse the sign for the last three components when constructing the index. Thus, the physical growth index is constructed so that larger values reflect more beneficial outcomes. For measurements during infancy, we use length instead of height and weight-for-length instead of BMI. We define underweight (overweight) as being below the 15th (above the 85th) percentile according the WHO weight-for-length growth standards (Organization et al., 2006) for infants less than one year old. For older children, we rely on the overweight and underweight measures developed in Cole et al. (2000) and implemented in Vidmar et al. (2004). Appendix Table A3 provides descriptive statistics on the physical growth measures for the control group.

We exclusively consider the height and weight instead of other anthropometric measurements, such as head circumference, for two reasons. First, audit test-retest correlations at age 6.5 were very high for height (0.84) and BMI (0.89) but substantially lower for head circumference (0.65) (Patel et al., 2013). Second, height and weight are consistently measured across all waves in contrast to other measurements. Moreover, we decided not to consider blood pressure, as audit test-retest correlations are particularly low (around 0.50); in results not reported, we do not find an effect on blood pressure, but the confidence intervals are large.

Infant Feeding During the routine health visits in wave 1, study pediatricians assessed infant feeding using standard questionnaires. Previous published studies have only reported the effect of the intervention on breastfeeding exclusivity and duration. We focus on maternal reports of the number of times and the total quantity of breast milk (including expressed and donor milk), infant formula, cow's milk (including other types of animal milk), water, juices or other liquids, and solid food (including cereals) the child received during the previous 24 hours at each visit. Less than 1.00 (0.01) percent of breast milk feedings are expressed (donor) milk, with the rest being breastfed at the breast. Thus, we refer to the combined group of breast milk as breastfeeding interchangeably. For breast milk and solid foods, mothers did not report the quantity but only the number

Table A3
Descriptive Statistics for the Control Group: Physical Growth Measures

	**Weight-for-Age** (1)	**Height-for-Age** (2)	**BMI-for-Age** (3)	**Underweight** (4)	**Overweight** (5)
1 Month	−0.10	0.10	−0.63	0.20	0.11
	(0.80)	(0.84)	(1.11)	(0.40)	(0.32)
2 Months	0.07	−0.00	−0.26	0.15	0.16
	(0.81)	(0.89)	(1.10)	(0.35)	(0.37)
3 Months	0.25	0.14	−0.06	0.12	0.17
	(0.83)	(0.94)	(1.08)	(0.33)	(0.38)
6 Months	0.45	0.23	0.54	0.03	0.34
	(0.87)	(1.00)	(0.98)	(0.17)	(0.47)
9 Months	0.47	0.28	0.81	0.01	0.50
	(0.84)	(0.95)	(0.90)	(0.09)	(0.50)
12 Months	0.51	0.34	0.93	0.00	0.59
	(0.84)	(0.91)	(0.89)	(0.07)	(0.49)
6 Years	0.04	0.21	−0.09	0.12	0.09
	(0.93)	(0.93)	(1.02)	(0.33)	(0.29)
11 Years	−0.01	0.26	−0.10	0.13	0.13
	(1.00)	(0.98)	(1.03)	(0.34)	(0.34)
16 Years	0.23	0.31	0.03	0.09	0.15
	(0.89)	(0.93)	(0.92)	(0.29)	(0.35)

Note: The table shows means and standard deviations (in parenthesis) for anthropometric measures from age 1 month to 16 years. Columns (1)-(3) show age-standardized z-scores for weight, height, and body mass index (BMI), while columns (4)-(5) report the share of children who are under- and overweight respectively. Underweight (overweight) measures are based on being below (above) the 15th (85th) percentile according to the WHO Child Growth Standards (WHO, 2020).

of times the infant received that type of food. We define a child as being exclusively breastfed when he or she only receives breast milk and nothing else.

We construct the infant feeding outcomes in Figure 3 as follows:

- *Calories from Formula* and *Calories from Cow's Milk* respectively indicate the calories the infant received from infant formula and cow's or other types of animal milk, calculated based on the quantity received and assuming that both types of liquid contain 65 kcal per 100 ml.

- *Calories from Juices* indicates the calories the infant received from juices and other liquids, assuming that two-thirds being apple juice (45 kcal per 100 ml) and one-third tea (0 kcal per 100 ml). From anecdotal evidence, *juices* would typically be apple juice and *other liquids* would be black tea.

- *Calories from Liquids (excl. BM)* is the total calorie intake from liquids (formula, cow's milk, and juices) excluding breast milk.

- *Calories/100 ml Liquids (excl. BM)* is constructed as *Calories from Liquids (excl. BM)* divided by the total volume of liquids excluding breast milk.

- *Total Volume Liquids (excl. BM), ml* indicates the total quantity of infant formula, cow's milk, water, juices, and other liquids measured in ml.

- *Volume of All Liquids (incl. BM)* indicates the total volume of liquids the child received including breast milk. For breastfed children at one to three months, we estimate breast milk intake based on the recommended daily calorie intake formula Calorie Intake $= 89 \times$ Weight in Kg $+ 75$ (Lupton et al., 2002), subtract their calorie intake from all other liquids, and calculate the volume assuming that breast milk contains 65 kcal per 100 ml. For breastfed children at 6 to 12 months, we assume that each breast milk feeding contains 175 ml.

- *Calories of All Liquids (incl. BM)* indicates the total calorie intake from liquids including breast milk, where calories from breast milk is estimated as described above.

For the mediation analysis in Section 5.3, we construct measures of the total cumulated calorie intake by respectively age one, two, and three months. To be precise, for the calculation of the total cumulated calorie intake at one month, we assume a calorie intake during the first month of life equal to the estimated calorie intake at the first month

health checkup multiplied by the age in days at this checkup. Similarly, we assume that the calorie intake during the second month is equal to the estimated calorie intake at the two months checkup multiplied by the age difference in days between the first and the second checkups. The total cumulated calorie intake at two months is thus the sum of the calorie intakes during the first and the second months. At age three months, we perform a similar calculation.

Infant Illness Index At each visit during infancy, pediatricians asked mothers to detail any episodes of skin rash, gastrointestinal illness, respiratory illness, other illness, and hospitalization since the previous visit. The infant illness index includes five indicators for whether the infant experienced any of the five outcomes at least once during infancy, and another five indicators representing multiple reports of the same outcome. In contrast to all other indices, a lower value on the infant illness index is better and indicates being less ill. The rates of illnesses during the first year of life were similar or slightly lower in our setting compared to reports from Canada, the United Kingdom, and the United States (Baker and Milligan, 2008; Fitzsimons and Vera-Hernández, 2022; Haider et al., 2014) (Appendix Tables A4).

Table A4
Descriptive Statistics for the Control Group: Infant Illnesses

	**Rash**	**Gastroenteritis Illness**	**Respiratory Illness**	**Other Illness**	**Hospitalization**
	(1)	(2)	(3)	(4)	(5)
Any	0.20	0.13	0.67	0.37	0.25
	(0.40)	(0.33)	(0.47)	(0.48)	(0.43)
Multiple	0.07	0.04	0.44	0.18	0.09
	(0.26)	(0.20)	(0.50)	(0.39)	(0.28)

Note: The table shows means and standard deviations (in parenthesis) for indicators of any and multiple episodes of the specific infant illness measure during the first year.

Cognitive Development We examine three dimensions of cognitive development. First, the academic index at age six includes teachers' ratings of each child's performance in reading, writing, and mathematics. Previous PROBIT research has shown large improvements in IQ at age 6.5, using the pediatrician administered Wechsler Abbreviated Scales of Intelligence (WASI) (Kramer et al., 2008*b*). However, given pediatricians were not

blind to treatment, we caution that the WASI measures may be associated with a potential measurement bias, as also pointed out by (Kramer et al., 2008*b*).[14] We therefore choose not to consider the WASI measures at age 6.5. Second, the IQ index at age 16 includes scores from the NeuroTrax cognitive tests for seven domains, measuring memory, executive function, visual–spatial perception, verbal function, attention, information processing, and fine motor skills. The tests were self-administered and computerized to minimize potential measurement bias caused by non-blinding of the pediatricians and within polyclinic correlations. Third, we consider whether the child is enrolled in the academic educational track at age 16. Finally, the cognitive index includes all eleven components of these three dimensions.

Academic Index At age six, teachers rated each child's academic performance in reading, writing, and mathematics on a five-point Likert scale as far below, somewhat below, at, somewhat above, or far above his or her grade level. We observe teacher ratings for about 60 percent of the children who are observed in the six year follow up; children without a teacher rating had not started school yet for the most part. Because of potential selection into the teacher sample as a consequence of the intervention, we choose to restrict the analysis sample to children who were supposed to have started school at the time they participated in the follow up assessment at the polyclinic. Children who had turned six years by September 1st were supposed to have started school that year. Within this restricted sample of children, 15.1 percent do not have a teacher assessment. As the main reason for not having a teacher assessment for this restricted sample is still not hav-

[14] At age 6, study pediatricians assessed children's cognitive development using the Wechsler Abbreviated Scales of Intelligence (WASI) —the best available IQ test at the time. The WASI consists of four subtests (vocabulary, similarities, block design, and matrix reasoning) and takes about 30 minutes to administer. One pediatrician assessed the children in 24 polyclinics and two did so in the remaining 7 high-volume polyclinics. The pediatricians were not blind to the treatment status, as they were involved in implementing the intervention. It would have been ideal to have had psychologists blind to the treatment status assess each child. However, that was practically impossible. Nowadays a second best solutions would have been to use computerized tests. But at the time of the assessment, neither the children nor the pediatricians knew how to use computers, and validated computer-assisted cognitive tests were not available. If they had used computerized tests, it could clearly have led to attenuation bias due to measurement error. Therefore, this was also not a possibility. Primarily due to the subjective nature of rating the child's responses, the WASI measures are associated with a high intraclass correlation coefficient (ICC) for cognitive scores of 0.31 (Kramer et al., 2008*b*). In the assessment, some pediatricians were very strict, while others were quite lenient. This led to a high variability in mean scores between polyclinics, even within each treatment group (Kramer et al., 2009). Kramer et al. (2008*b*) conducted an audit of 190 children. They found the lowest test-retest correlations for verbal IQ (vocabulary and similarities) of 0.62, for which the scope for subjective assessment of the child might be largest, compared to correlations of 0.71 in performance IQ (block design and matrix reasoning).

ing started school, we assume that the reason for not being in school is that they were not school ready. We therefore impute their teacher rating with "below" their grade level, where we combine the teacher ratings "far below" and "somewhat below" to reduce the skewness of the distribution, as less than three percent of the graded children have a grade of far below. Appendix Figure A6 shows that our results are robust to alternative constructions of the academic index, both in terms of the restriction of the sample and the imputation. Teachers were unaware of the children's participation in the study (and therefore also of their treatment status) and had only three to four PROBIT children on average (Kramer et al., 2008*a*). Consequently, any potential bias in the teacher reports should be unrelated to the treatment status.

IQ Index At age 16, children's neurocognitive function was assessed using a computerized battery of the NeuroTrax cognitive tests. The tests were self-administered to minimize potential measurement bias caused by non-blinding of the pediatricians and within polyclinic correlations, resulting in ICCs as small as 0.02 (Yang et al., 2018). The test battery consists of 10 short subtests that assess both verbal and nonverbal domains of cognitive function. From this, we have standardized scores for seven domains, measuring memory, executive function, visual–spatial perception, verbal function, attention, information processing, and fine motor skills. We construct a cognitive skills index, based on these seven scores, to reflect general neurocognitive function.

Academic Track The academic track indicated whether the child is enrolled in the academic educational track at age 16 (1) or not (0).

Cognitive Index The cognitive index includes the three components from the academic index, the seven components from the IQ index, and the academic track.

Socioemotional Skills Index The socioemotional skills summary index includes seven individual components. The measures are constructed based on parent reports at child age six to the Strengths and Difficulties Questionnaire (SDQ) and to supplemental behavioral questions taken from the Canadian National Longitudinal Survey of Children and Youth (NLSCY). Using the SDQ answers, we construct the five standard subscales for emotional symptoms, conduct problems, hyperactivity/inattention, peer relationship problems, and prosocial behavior; we reverse the sign of the first four subscales so that

Figure A6
The Effect of Breastfeeding Promotion on Alternative Measures of Teacher's Academic Assessment

(a) Academic Index

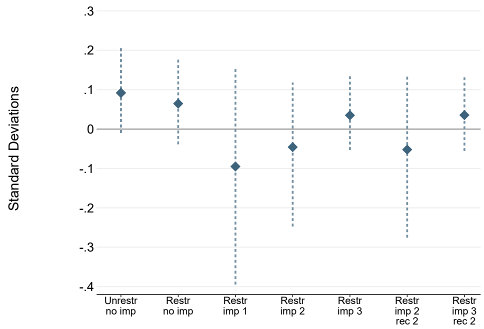

(b) Math

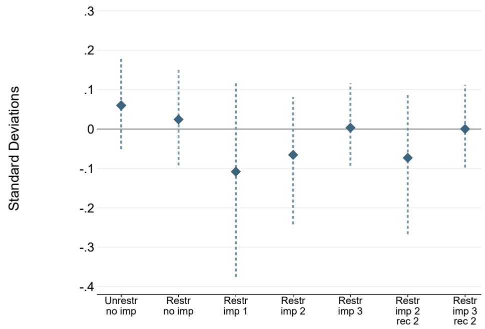

(c) Reading

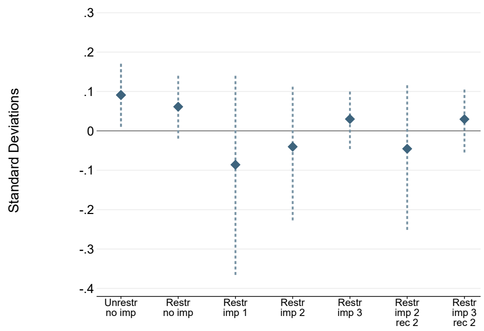

(d) Writing

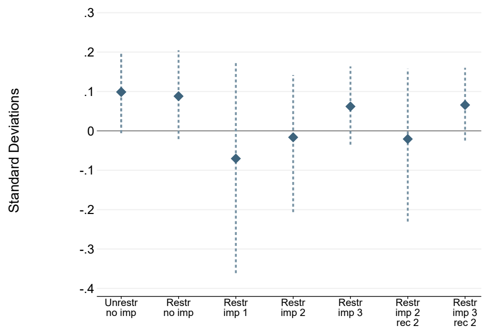

Note: Teachers rated each child's academic performance in reading, writing, and mathematics on a five-point Likert scale as far below (1), somewhat below (2), at (3), somewhat above (4), or far above (5) his or her grade level. All outcome variables are stanradized so the control group has a mean of zero and a standard deviation of one. The sample is restricted to all children with at least one measurement in wave 2. The restricted sample only includes children who were supposed to be in school. The first of the seven estimates is without this restriction. The second estimate comes from the restricted sample without any imputation. From the third to the fifth estimate, missing values in this restricted sample are imputed with far below grade (1), somewhat below grade (2), or at grade level (3), respectively. The sixth and seventh estimates additionally recode far below grade (1) with somewhat below grade (2) and impute missing values in this restricted sample with somewhat below grade (2) and at grade level (3) respectively. Each estimate comes from a separate regression as specified in equation (1); the dashed lines show the 95 percent confidence interval based on wild cluster bootstrapped (WCB) standard errors clustered at the hospital level. Multiple hypothesis testing using the *krieger* method is performed on all estimates within the same graph. Significance levels after testing for multiple hypothesis are indicated as follows: $\circ p < 0.10$, $\bullet p < 0.05$, $*p < 0.01$.

a higher score is more favorable. Based on the questions from the NLSCY, we construct two summary indices capturing respectively externalizing (five questions) and internalizing (ten questions) behavioral problems; we reverse the sign of these measures so that a higher score is more favorable. The summary index includes the seven components: the five SDQ subscales and the two NLSCY behavioral problem indices.

A.4 Original Analysis Plan

When applying for access to the PROBIT data, we included a study protocol outlining our plan for the empirical analysis. Our work commenced with three original aims that revealed null or no novel results. For transparency, we here describe the original three aims and include the results in the Appendix.

First, we planned to construct summary indices that aggregate information over multiple treatment effect estimates —in particular, summary indices of health (Appendix Figures A7e and A8a) and human capital (Appendix Figures A9a and A9d). We had also planned to consider a parental investment index; however, the data proved not to provide sufficient variation on parental inputs. Second, we proposed exploring heterogeneity in the effects of breastfeeding by socioeconomic status and child gender (see Appendix Section A.6). Third, to convince (particularly) economists about the validity of a causal interpretation of the intervention, we planned to provide extensive randomization tests (Appendix Table A1). In terms of evaluating the breastfeeding intervention, our primary plan was to estimate the intention to treat (ITT) effects, as done in the main analysis (see Section 3). We also mentioned that we would scale the magnitude of the effects of exclusive (or any) breastfeeding for at least 3 and 6 months respectively by using the random assignment of treatment as an instrumental variable (IV). However, this analysis did not provide any additional insights for two reasons. First, the point estimates using IV are even more noisily estimated than for ITT, as shown in existing PROBIT papers (Oken et al., 2013; Owen et al., 2018; Martin et al., 2013, 2017; Yang et al., 2018). Second, given the results from our infant feeding analysis, we are not convinced that the exclusion restriction is valid in this particular context.

A.5 Effects on Child Health and Development

In this appendix section, we report results on child health and development. Overall, all results are in line with previous PROBIT research and we therefore chose not to include

Figure A7
The Effect of Breastfeeding Promotion on Physical Growth

(a) Height-for-age

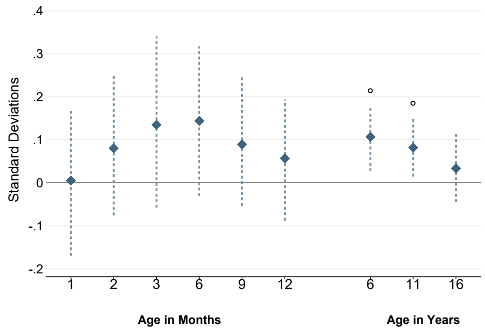

(b) Weight-for-Length / BMI

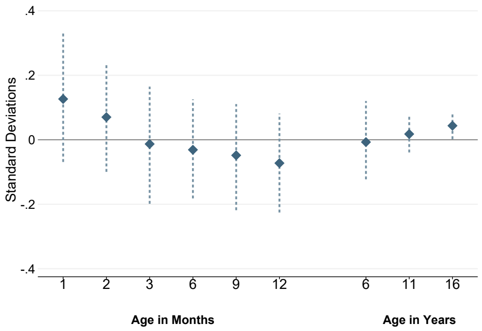

(c) Probability of Underweight

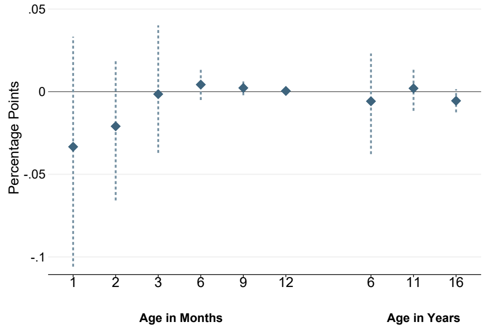

(d) Probability of Overweight

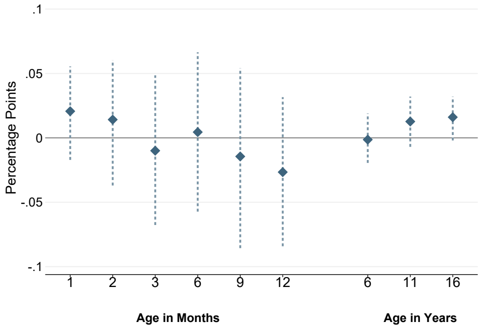

(e) Physical Growth Index

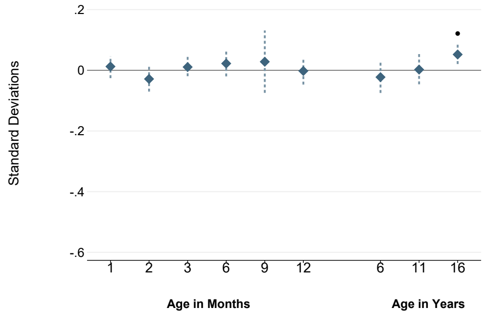

Note: Each estimate comes from a separate regression as specified in equation (1); the dashed lines show the 95 percent confidence interval based on wild cluster bootstrapped (WCB) standard errors clustered at the hospital level. Multiple hypothesis testing using the *krieger* method is performed on all estimates within the same graph. Significance levels after testing for multiple hypothesis are indicated as follows: ○$p < 0.10$, ●$p < 0.05$, *$p < 0.01$.

them in the main paper for brevity.

Appendix Figure A7 shows the effects of the breastfeeding promotion intervention on physical growth from age one month throughout childhood. Overall, we do not find consistent or persistent effects on height-for-age, weight-for-length/BMI, the probability of being overweight or underweight, or the physical growth index. These results are overall consistent with previous findings from the PROBIT study (Kramer et al., 2002, 2007; Martin et al., 2013, 2017). In contrast to Martin et al. (2017), however, we do not find evidence of an increased probability of being overweight. The estimate of the intervention on the probability of being overweight at age 16 is 1.61 percentage points with a 95 percent confidence interval [-0.20; 3.21] (see Appendix Figure A7d). This corresponds to an increased risk of being overweight by 11 percent compared to the control group, which is comparable to the estimate of 14 percent (CI [2; 28]) found in Martin et al. (2017). The main reason for us finding a statistically insignificant effect compared to Martin et al. (2017) is the difference in the way of clustering the standard errors. In the main text, we find a statistically significant and persistent effect of the intervention on weight-for-age (Figure 1). In infancy, this is in line with Kramer et al. (2002) who find positive effects on weight at ages 3, 6, and 9 months, but not at age 12 months as we also do not find. During childhood, Martin et al. (2017) find positive effects on weight gain from age 2.8 to 14.5 years (Table 3) and on weight at ages 6.5, 11.5, and 16.2 (Figure 2A) —again in line with our findings.

Appendix Figure A8 shows the effects of the breastfeeding promotion intervention on infant illness. Although our summary index and its individual components are not statistically significant, the estimates suggest that the breastfeeding intervention decreased the likelihood of infant illness. The point estimates are all negative and we cannot rule out medium-sized reductions in illness of up to 0.17-0.48 standard deviations. For example, the point estimate for the illness index is -0.21 (p-value$=0.16$) and its 95 percent confidence interval ranges from -0.48 to 0.09 standard deviations. The point estimates for rash and gastroenteritis are -0.15 (95 percent confidence interval not adjusted for multiple hypothesis testing $[-0.30; -0.01]$) and -0.13 ($[-0.25; 0.01]$) standard deviations, respectively. We do not find any heterogeneity in the effects on dimensions proxying for the infectious environment; Appendix Figure A8b shows no meaningful differences across subgroups split by parity and season of birth. Overall, the results are consistent with Kramer et al. (2001), finding negative effects on the risk of any gastrointestinal tract infection, atopic eczema, and rash in the first 12 months. Despite

Figure A8
The Effect of Breastfeeding Promotion on Infant Illness

(a) Main effects **(b)** Heterogeneity by Infectious Environment

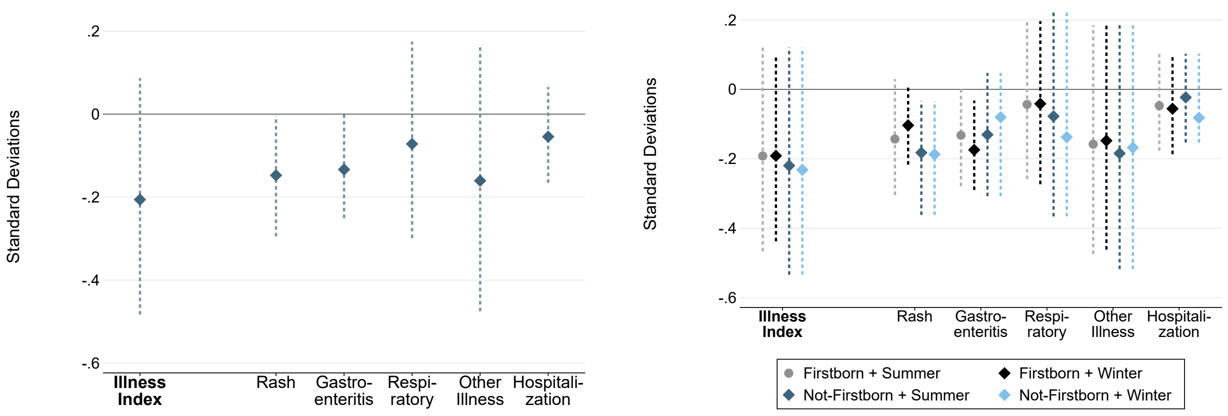

Note: Each estimate comes from a separate regression as specified in equation (1); the dashed lines show the 95 percent confidence interval based on wild cluster bootstrapped (WCB) standard errors clustered at the hospital level. The models in graph A8a also control for indicators of age at last visit in months and the total number of observations during wave 1. Multiple hypothesis testing using the *krieger* method is performed on all estimates within the same graph. Significance levels after testing for multiple hypothesis are indicated as follows: $\circ p < 0.10$, $\bullet p < 0.05$, $*p < 0.01$.

differences in precision, Kramer et al. (2001)'s and our effect sizes for rash (-36 vs. -29 percent) and gastroenteritis (-38 vs. -34 percent) are relatively similar when expressed as percent changes.

Consistent with (Kramer et al., 2008*b*), we do not find statistically significant effects on teachers' assessment of children's academic performance at age 6 (Appendix Figure A9b). Consistent with (Yang et al., 2018), we do not find statistically significant effects on IQ at age 16 (Appendix Figure A9c). The overall cognitive index also shows little statistical evidence of effects (Graph A9a) with a point estimate of 0.02 standard deviation—though with a wide 95 percent confidence interval ranging from -0.24 to 0.28. Consistent with (Kramer et al., 2008*a*), we also do not find statistically significant effects on children's socioemotional skills at age 6 (Appendix Figure A9d).

We find little evidence that this breastfeeding intervention has significant effects on our measures of cognitive development in Belarus, although such effects are theoretically possible. Any effect of breastfeeding on cognitive development is typically hypothesized to be due to changes in the social stimulation of the child or due to the particular composition of breast milk (Fitzsimons and Vera-Hernández, 2022). The most important nutritional difference between formula and breast milk is breast milk's content of two long-chain polyunsaturated fatty acids—docosahexaenoic acid (DHA) and arachi-

Figure A9
The Effect of Breastfeeding Promotion on Cognitive and Socioemotional Development

(a) Cognitive Development

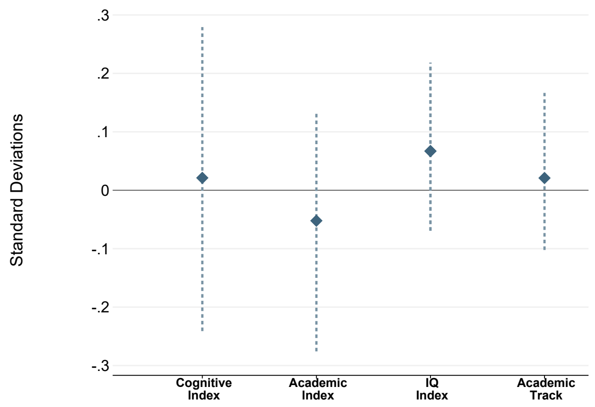

(b) Academic at Age 6

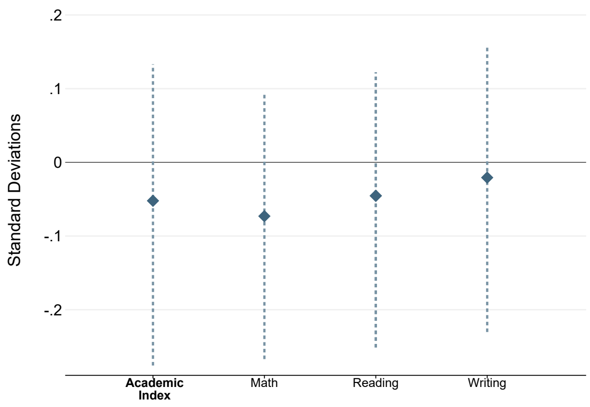

(c) IQ at Age 16

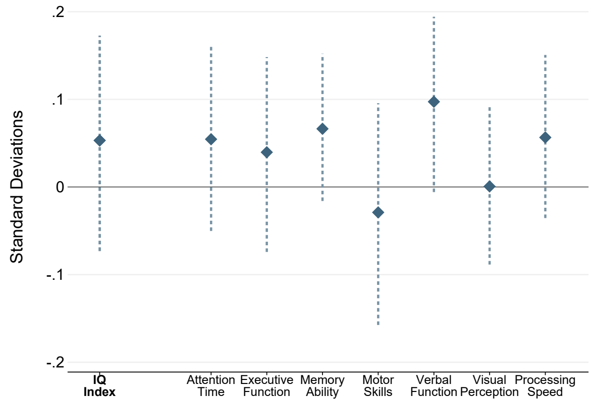

(d) Socioemotional Skills at Age 6

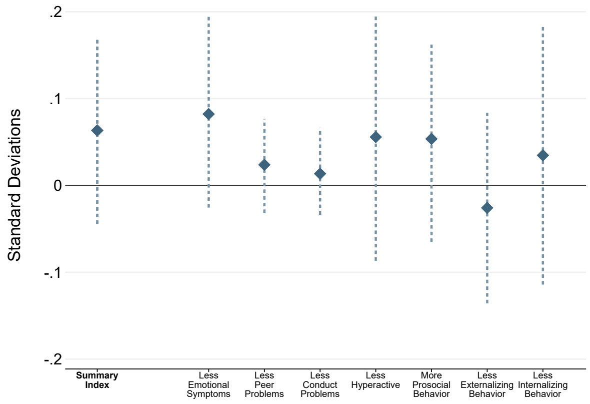

Note: Each estimate comes from a separate regression as specified in equation (1); the dashed lines show the 95 percent confidence interval based on wild cluster bootstrapped (WCB) standard errors clustered at the hospital level. Multiple hypothesis testing using the *krieger* method is performed on all estimates within the same graph. Significance levels after testing for multiple hypothesis are indicated as follows: ∘$p < 0.10$, •$p < 0.05$, *$p < 0.01$.

donic acid (ARA)—that are crucial for infants' neurocognitive development (Krol and Grossmann, 2018). However, infants produce insufficient amounts of these fatty acids (Cockburn, 2003). This is the reason for adding DHA and ARA to infant formula, which is standard in high-quality infant formula today, but was not the case in our setting nor in the one of Fitzsimons and Vera-Hernández (2022)—the only other published causal study of breastfeeding on cognitive development outside of the PROBIT RCT.[15]

[15]Since 1996 (2002), the European Union (United States) has allowed the addition of DHA and ARA to

But in contrast to our results, Fitzsimons and Vera-Hernández (2022) find beneficial effects on cognitive skills among children of low educated mothers at ages 3–7 in the United Kingdom. In their investigation of mechanisms, they do not find robust effects on child health, socioemotional skills, or any factors related to maternal investments or attachment. This suggests that the positive effect of breastfeeding on cognitive skills in their setting could be due to the nutritional composition of breast milk rather than changes in the social environment. One key difference between these two settings is the prevalence of any breastfeeding among the control group, and thereby the intake of some quantity of DHA and ARA. Most mothers in Fitzsimons and Vera-Hernández (2022) only breastfed (partially) for a few weeks or did not initiate breastfeeding at all, instead relying on almost exclusive use of infant formula without added DHA and ARA. In contrast, most control mothers in Belarus partially breastfed for at least three months. As seen in Figure 2, mothers in the control group still breastfed an average of 3.5 times per day at three months, so infants still ingested some amount of these fatty acids through breast milk.

Our results suggest that increasing the frequency of breastfeeding and the duration of breastfeeding exclusivity does not improve cognitive development. This result is important for policy discussions about the optimal duration of maternity leave and lactation policies at the workplace. The results also suggest that adding DHA and ARA to infant formula might remove or at least substantially reduce any beneficial effect of breastfeeding on cognitive development among healthy infants (Cockburn, 2003; Hoffman, Boettcher and Diersen-Schade, 2009; Lien, Richard and Hoffman, 2018). While more causal work is needed before drawing strong conclusions, this would have important policy implications because governments regulate the composition of infant formula. To the extent that these additives are much more commonly found in high-quality infant formula today, our estimates of the benefits of breastfeeding may reasonably represent an upper bound of any potential beneficial effect of breastfeeding on cognitive development in settings where high-quality infant formula is widely used.

formula and made the addition of DHA mandatory in 2020.

A.6 Heterogeneity

In this appendix, we report all the results when allowing for heterogeneous response on two separate dimensions. First, we consider whether the treatment effects differ by gender. Second, we consider whether the treatment effects differ by socioeconomic status (SES). We define socioeconomic disadvantage to be the case when neither of the parents has a university degree nor works in a non-manual occupation (services), meaning the group of all other parents represents socioeconomic advantage.

To estimate heterogeneous effects, we modify the empirical specification given in equation (1). We estimate fully interacted models, in which we interact all control variables with the particular dimensions of interest. For the estimation of heterogeneity with respect to gender, the specification is the following (with the model for SES being completely analogous):

$$\begin{aligned} Y_{iph} =& \gamma_0^{female} Female + \gamma_1^{female} Treatment_h \times Female + \gamma_1^{male} Treatment_h \times Male + \\ & Female \times Z_i' \delta^{female} + Male \times Z_i' \delta^{male} + \theta_p^{female} + \theta_p^{male} + \varepsilon_{iph}, \end{aligned} \tag{4}$$

where *Female* (*Male*) takes the value 1 (0) for girls and 0 (1) for boys. The remaining variables are similar to those specified for specification (1).

As shown in the following figures, there is little evidence of meaningful heterogeneity in any of the outcomes we study by either gender or SES.

Figure A10

The Effect of Breastfeeding Promotion on Infant Illness and Childhood Physical Growth: Heterogeneity by Gender

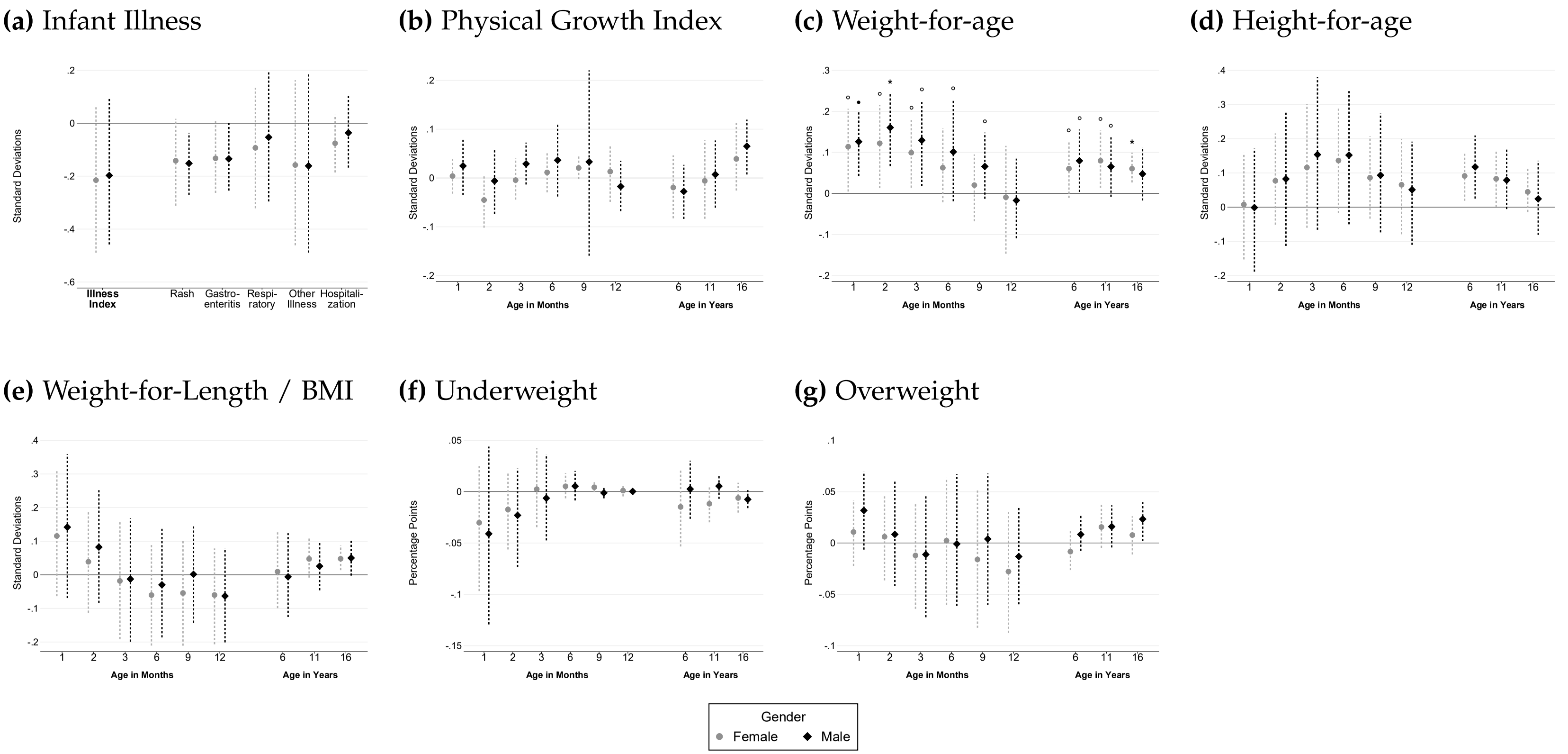

Note: Each set of estimates comes from a separate regression as specified in equation (4); the dashed lines show the 95 percent confidence interval based on wild cluster bootstrapped (WCB) standard errors clustered at the hospital level. Multiple hypothesis testing using the *krieger* method is performed on all estimates within the same graph. Significance levels after testing for multiple hypothesis are indicated as follows: $\circ p < 0.10$, $\bullet p < 0.05$, $*p < 0.01$.

Figure A11
The Effect of Breastfeeding Promotion on Infant Illness and Childhood Physical Growth: Heterogeneity by Socioeconomic Status

(a) Infant Illness

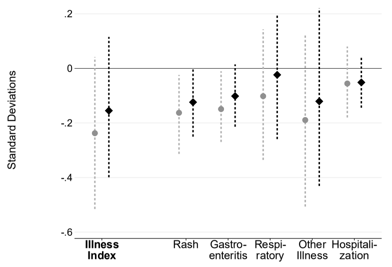

(b) Anthropometric Index

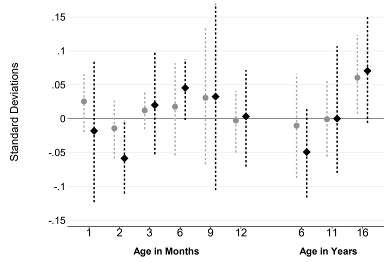

(c) Weight-for-age

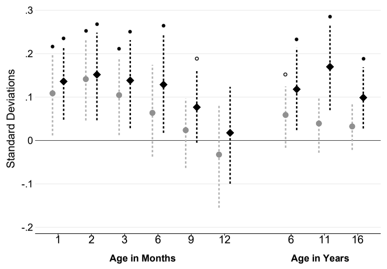

(d) Height-for-age

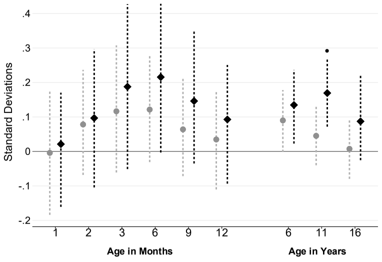

(e) Weight-for-Length / BMI

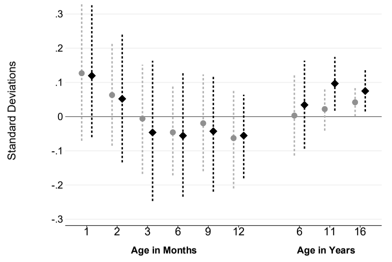

(f) Underweight

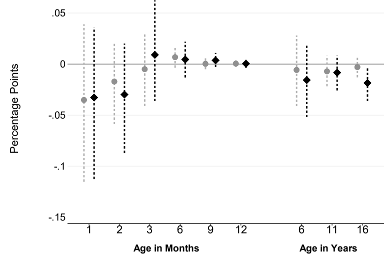

(g) Overweight

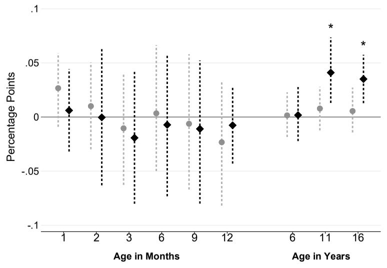

Socio-economic status
● Advantaged ◆ Disadvantaged

Note: Each set of estimates comes from a separate regression as specified in equation (4); the dashed lines show the 95 percent confidence interval based on wild cluster bootstrapped (WCB) standard errors clustered at the hospital level. Multiple hypothesis testing using the *krieger* method is performed on all estimates within the same graph. Significance levels after testing for multiple hypothesis are indicated as follows: $\circ p < 0.10$, $\bullet p < 0.05$, $*p < 0.01$.

Figure A12

The Effect of Breastfeeding Promotion on Cognitive Development: Heterogeneity by Gender and Socioeconomic Status

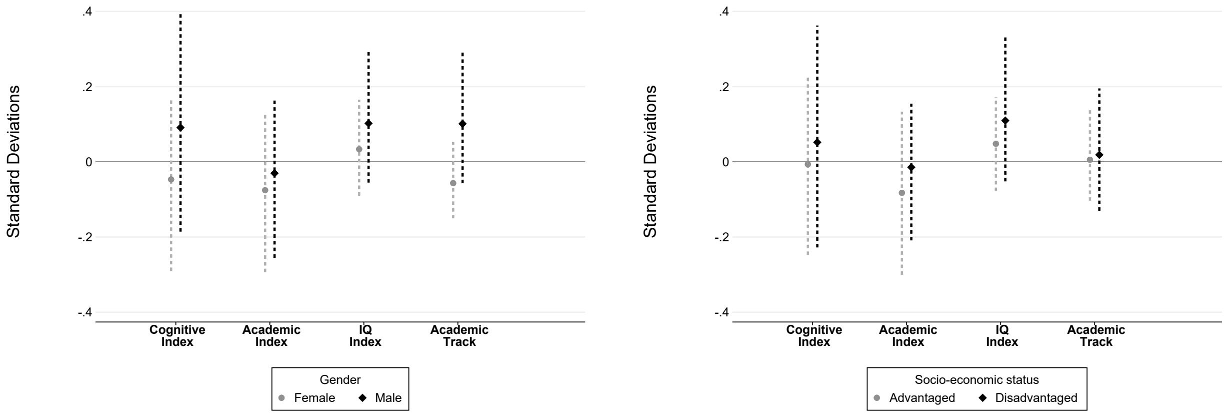

Note: Each set of estimates comes from a separate regression as specified in equation (4); the dashed lines show the 95 percent confidence interval based on wild cluster bootstrapped (WCB) standard errors clustered at the hospital level. Multiple hypothesis testing using the *krieger* method is performed on all estimates within the same graph. Significance levels after testing for multiple hypothesis are indicated as follows: $\circ p < 0.10$, $\bullet p < 0.05$, $^{*}p < 0.01$.

Figure A13
The Effect of Breastfeeding Promotion on Socialemotional Skills:
Heterogeneity by Gender and Socioeconomic Status

(a) Parent-reported, by Gender

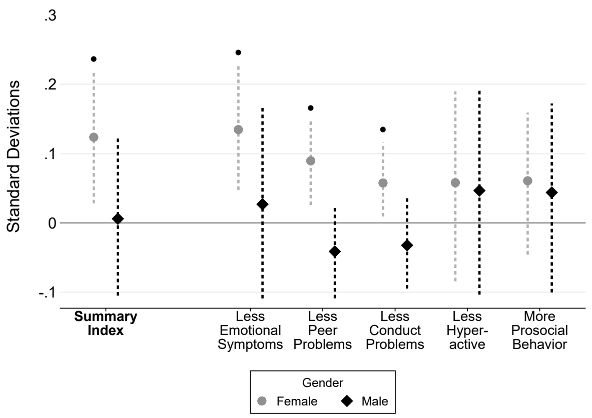

(b) Parent-reported, by SES

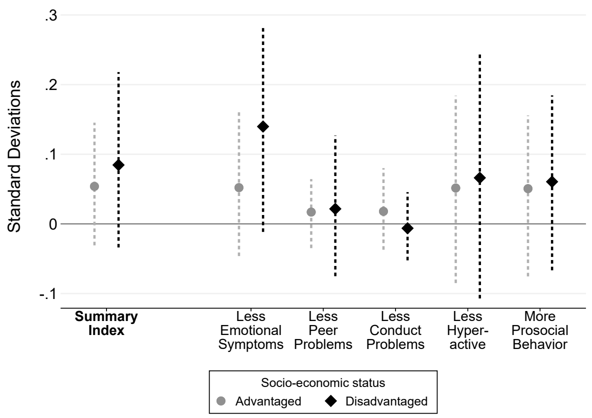

(c) Teacher-reported, by Gender

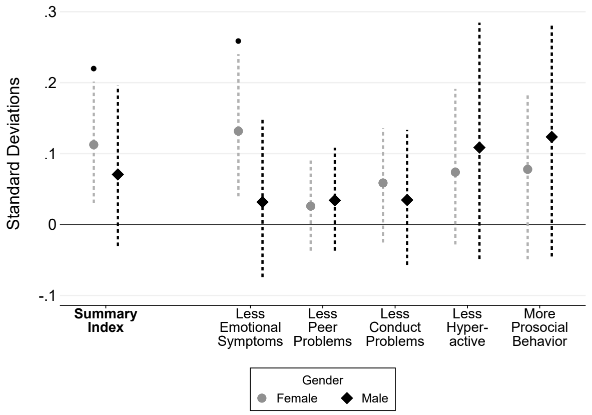

(d) Teacher-reported, by SES

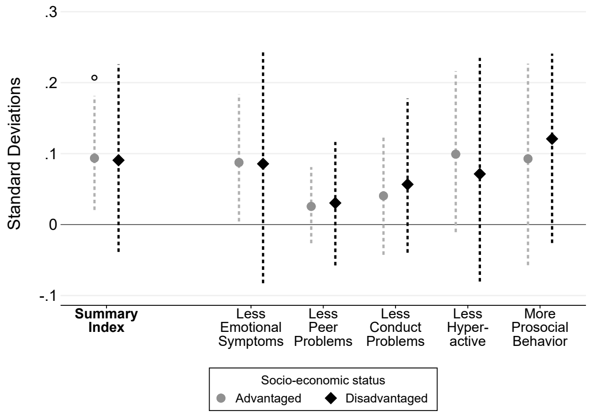

Note: Each set of estimates comes from a separate regression as specified in equation (4); the dashed lines show the 95 percent confidence interval based on wild cluster bootstrapped (WCB) standard errors clustered at the hospital level. Multiple hypothesis testing using the *krieger* method is performed on all estimates within the same graph. Significance levels after testing for multiple hypothesis are indicated as follows: $\circ p < 0.10$, $\bullet p < 0.05$, $^{*}p < 0.01$.

Figure A14

The Effect of Breastfeeding Promotion on Infant Feedings: Heterogeneity by Gender

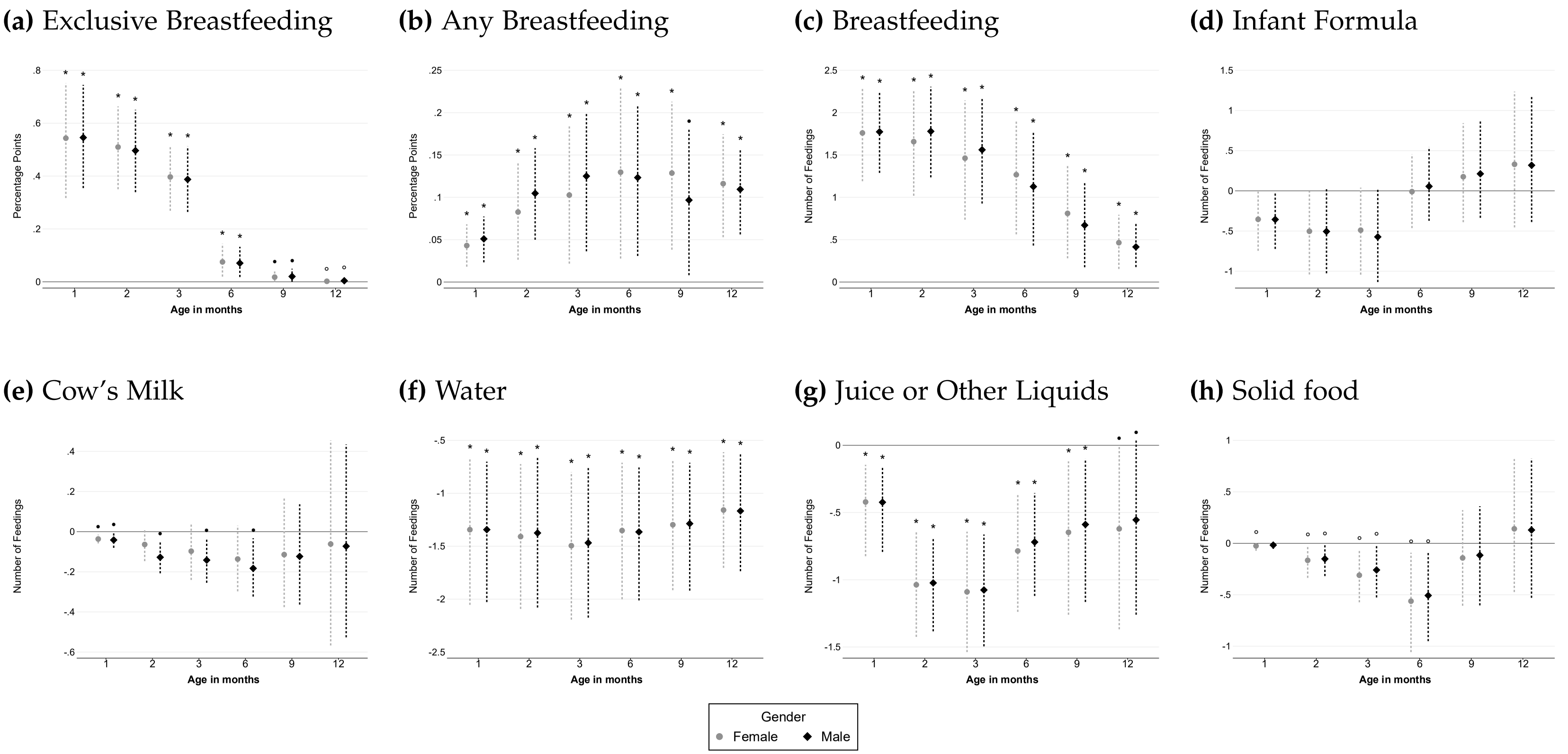

Note: Each set of estimates comes from a separate regression as specified in equation (4); the dashed lines show the 95 percent confidence interval based on wild cluster bootstrapped (WCB) standard errors clustered at the hospital level. Multiple hypothesis testing using the *krieger* method is performed on all estimates within the same graph. Significance levels after testing for multiple hypothesis are indicated as follows: $\circ p < 0.10$, $\bullet p < 0.05$, $*p < 0.01$.

Figure A15
The Effect of Breastfeeding Promotion on Infant Feedings: Heterogeneity by Socioeconomic Status

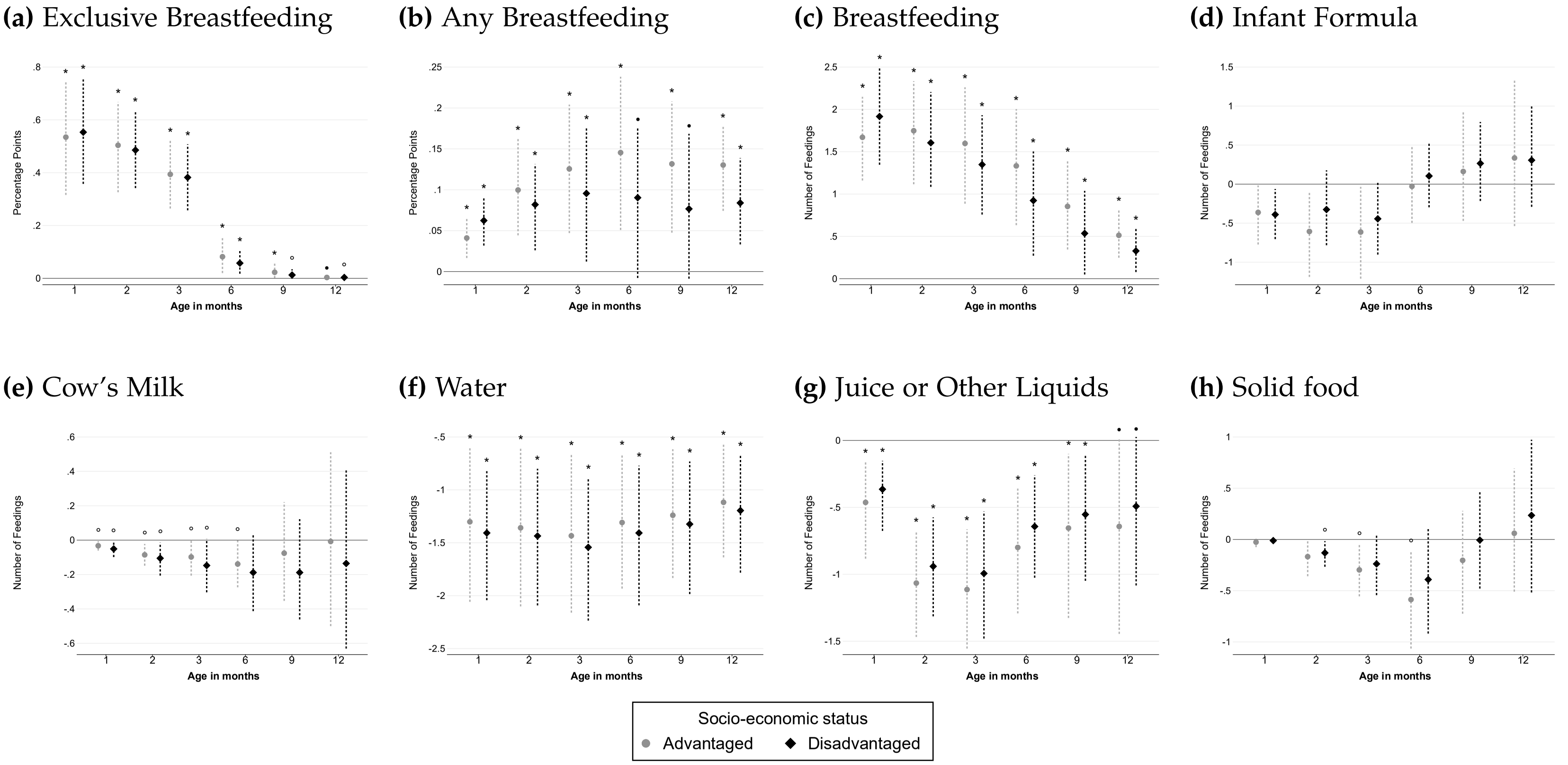

Note: Each set of estimates comes from a separate regression as specified in equation (4); the dashed lines show the 95 percent confidence interval based on wild cluster bootstrapped (WCB) standard errors clustered at the hospital level. Multiple hypothesis testing using the *krieger* method is performed on all estimates within the same graph. Significance levels after testing for multiple hypothesis are indicated as follows: $\circ p < 0.10$, $\bullet p < 0.05$, $^{*}p < 0.01$.

Figure A16

The Effect of Breastfeeding Promotion on Estimated Infant Liquid Calorie Intake, excluding Breast Milk (BM): Heterogeneity by Gender

(a) Calories from Formula

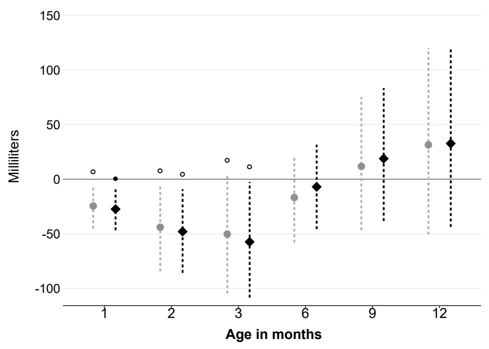

(b) Calories from Cow's Milk

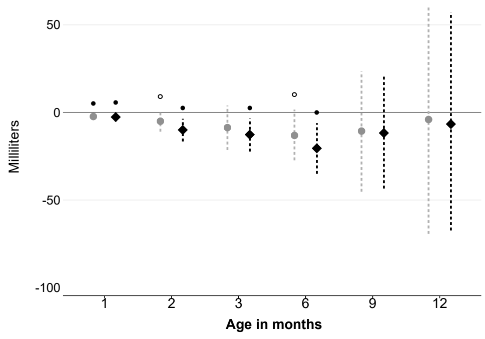

(c) Calories from Juices

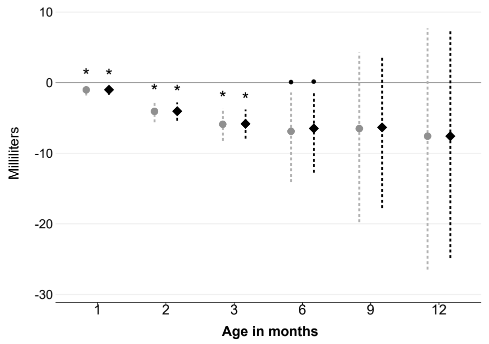

(d) Calories from Liquids (excl. BM)

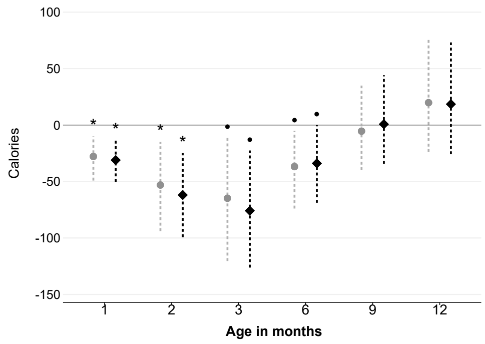

(e) Volume of Water

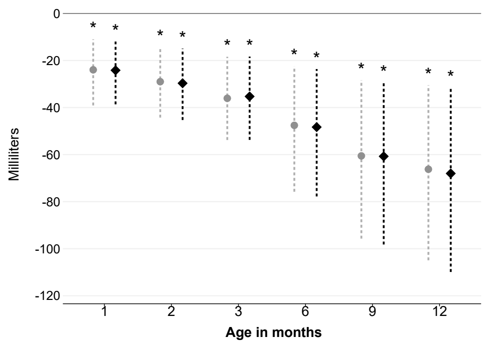

(f) Calories/100 ml Liquids (excl. BM)

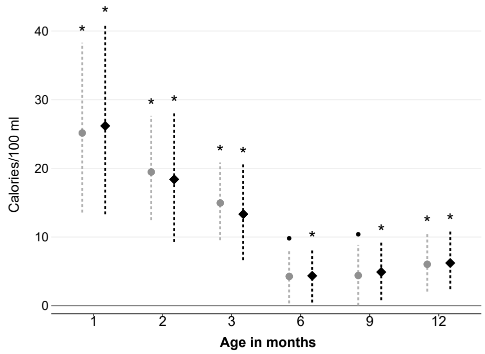

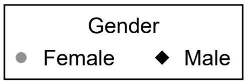

Note: Each set of estimates comes from a separate regression as specified in equation (4); the dashed lines show the 95 percent confidence interval based on wild cluster bootstrapped (WCB) standard errors clustered at the hospital level. Multiple hypothesis testing using the *krieger* method is performed on all estimates within the same graph. Significance levels after testing for multiple hypothesis are indicated as follows: $\circ p < 0.10$, $\bullet p < 0.05$, $^{*}p < 0.01$.

Figure A17

The Effect of Breastfeeding Promotion on Estimated Infant Liquid Calorie Intake, excluding Breast Milk (BM): Heterogeneity by Socioeconomic Status

(a) Calories from Formula

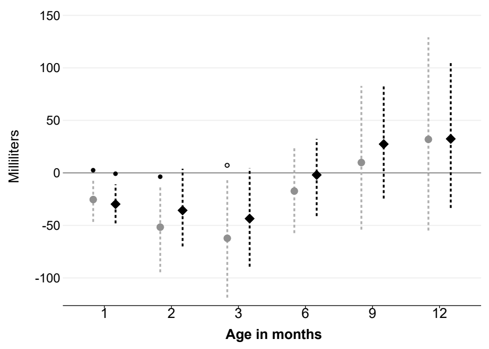

(b) Calories from Cow's Milk

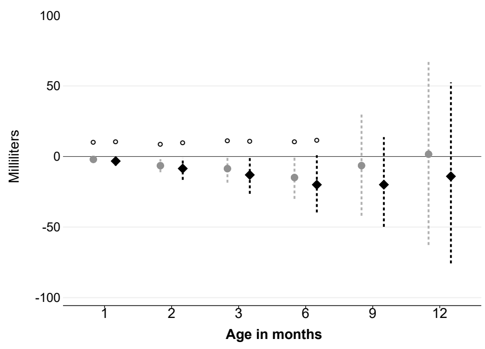

(c) Calories from Juices

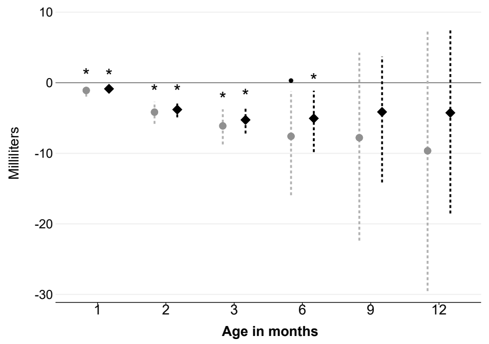

(d) Calories from Liquids (excl. BM)

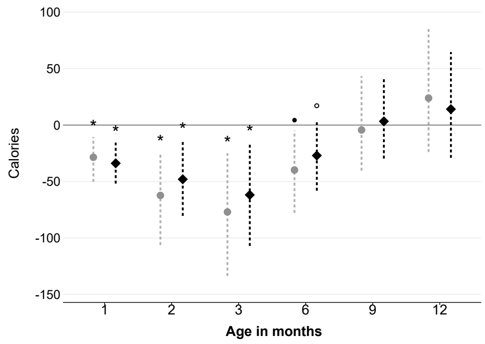

(e) Volume of Water

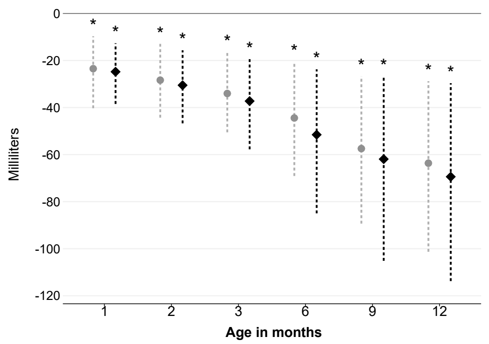

(f) Calories/100 ml Liquids (excl. BM)

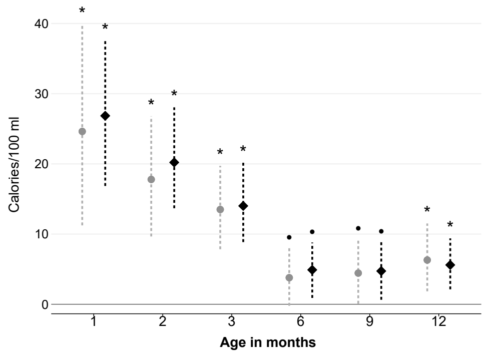

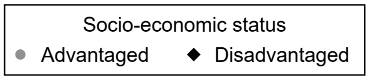

Note: Each set of estimates comes from a separate regression as specified in equation (4); the dashed lines show the 95 percent confidence interval based on wild cluster bootstrapped (WCB) standard errors clustered at the hospital level. Multiple hypothesis testing using the *krieger* method is performed on all estimates within the same graph. Significance levels after testing for multiple hypothesis are indicated as follows: ∘$p < 0.10$, •$p < 0.05$, *$p < 0.01$.

Figure A18

The Effect of Breastfeeding Promotion on Estimated Infant Liquid Calorie Intake, including Breast Milk: Heterogeneity by Gender

(a) Total Volume Liquids (excl. BM)

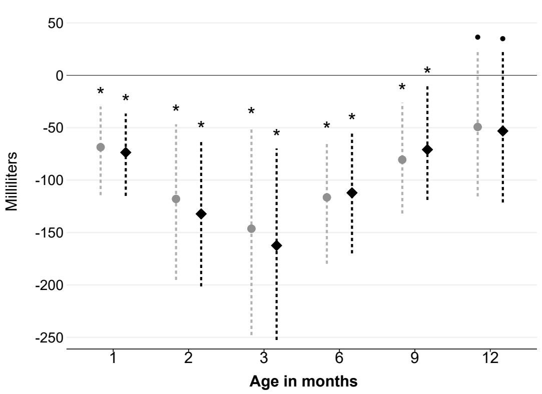

(b) Volume of All Liquids (incl. BM)

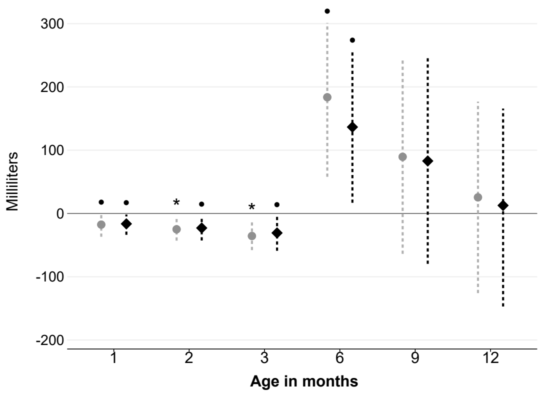

(c) Calories of All Liquids (incl. BM)

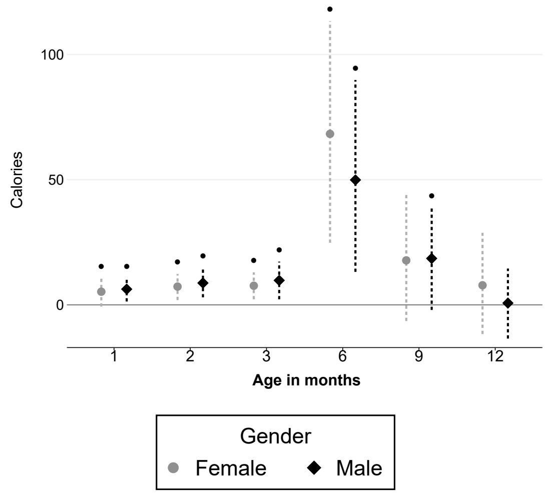

Note: Each set of estimates comes from a separate regression as specified in equation (4); the dashed lines show the 95 percent confidence interval based on wild cluster bootstrapped (WCB) standard errors clustered at the hospital level. Multiple hypothesis testing using the *krieger* method is performed on all estimates within the same graph. Significance levels after testing for multiple hypothesis are indicated as follows: ∘$p < 0.10$, •$p < 0.05$, *$p < 0.01$.

Figure A19
The Effect of Breastfeeding Promotion on Estimated Infant Liquid Calorie Intake, including Breast Milk: Heterogeneity by Socioeconomic Status

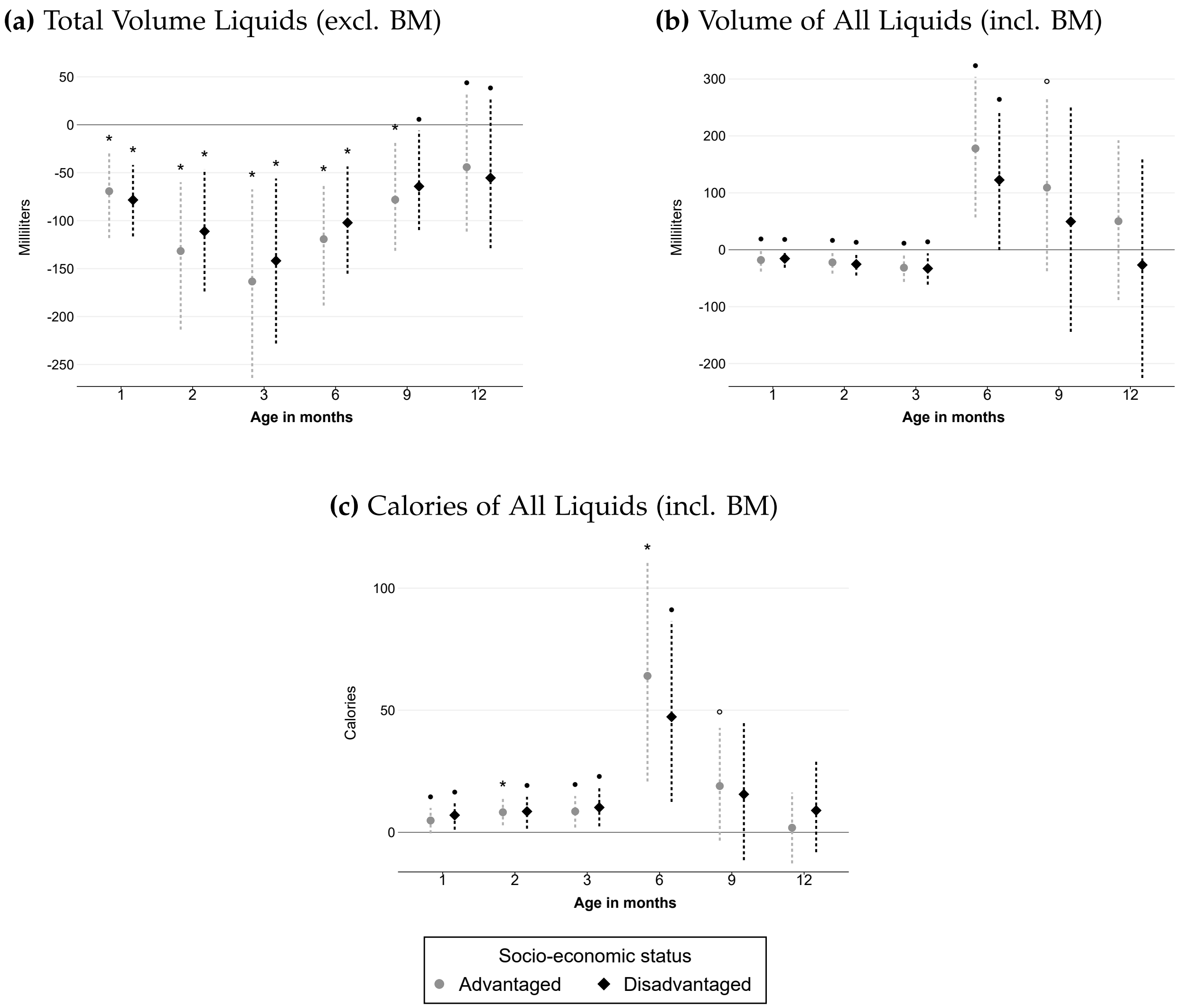

Note: Each set of estimates comes from a separate regression as specified in equation (4); the dashed lines show the 95 percent confidence interval based on wild cluster bootstrapped (WCB) standard errors clustered at the hospital level. Multiple hypothesis testing using the *krieger* method is performed on all estimates within the same graph. Significance levels after testing for multiple hypothesis are indicated as follows: ○$p < 0.10$, ●$p < 0.05$, *$p < 0.01$.

A.7 Institutional review board approval

PROBIT I and II were approved by the Belarusian Ministry of Health and received ethical approval from the McGill University Health Centre Research Ethics Board; PROBIT III and IV were approved by the Belarusian Ministry of Health and received ethical approval from the McGill University Health Centre Research Ethics Board, the Human Subjects Committee at Harvard Pilgrim Health Care, and the Avon Longitudinal Study of Parents and Children (ALSPAC) Law and Ethics Committee. A parent or legal guardian provided written informed consent in Russian at enrollment and at the follow-up visits, and all children provided written assent at the 11.5-year and 16-year visit.

A.8 Acknowledgments

The Human Subjects Committee of the Faculty of Economics, Business Administration and Information Technology at the University of Zurich has authorized the research project (OEC IRB # 2018-039).

References

Anderson, Michael L. 2008. "Multiple inference and gender differences in the effects of early intervention: A reevaluation of the Abecedarian, Perry Preschool, and Early Training Projects." *Journal of the American statistical Association*, 103(484): 1481–1495.

Baker, Michael, and Kevin Milligan. 2008. "Maternal employment, breastfeeding, and health: Evidence from maternity leave mandates." *Journal of health economics*, 27(4): 871–887.

Cockburn, F. 2003. "Role of infant dietary long-chain polyunsaturated fatty acids, liposoluble vitamins, cholesterol and lecithin on psychomotor development." *Acta Paediatrica*, 92: 19–33.

Cole, Tim J, Mary C Bellizzi, Katherine M Flegal, and William H Dietz. 2000. "Establishing a standard definition for child overweight and obesity worldwide: international survey." *Bmj*, 320(7244): 1240.

Fitzsimons, Emla, and Marcos Vera-Hernández. 2022. "Breastfeeding and child development." *American Economic Journal: Applied Economics*, 14(3): 329–366.

Haider, Steven J, Lenisa V Chang, Tracie A Bolton, Jonathan G Gold, and Beth H Olson. 2014. "An evaluation of the effects of a breastfeeding support program on health outcomes." *Health services research*, 49(6): 2017–2034.

Hoffman, Dennis R, Julia A Boettcher, and Deborah A Diersen-Schade. 2009. "Toward optimizing vision and cognition in term infants by dietary docosahexaenoic and arachidonic acid supplementation: a review of randomized controlled trials." *Prostaglandins, Leukotrienes and essential fatty acids*, 81(2-3): 151–158.

Kling, Jeffrey R., Jeffrey B. Liebman, and Lawrence F. Katz. 2007. "Experimental Analysis of Neighborhood Effects." *Econometrica*, 75(1): 83–119.

Kramer, Michael S, Beverley Chalmers, Ellen D Hodnett, Zinaida Sevkovskaya, Irina Dzikovich, Stanley Shapiro, Jean-Paul Collet, Irina Vanilovich, Irina Mezen, Thierry Ducruet, George Shishko, Vyacheslav Zubovich, Dimitri Mknuik, Elena Gluchanina, Viktor Dombrovskiy, Anatoly Ustinovitch, Tamara Kot, Natalia Bogdanovich, Lydia Ovchinikova, and Elisabet Helsing. 2000. "PROMOTION OF BREASTFEEDING INTERVENTION TRIAL (PROBIT): A CLUSTER-RANDOMIZED TRIAL IN THE REPUBLIC OF BELARUS." *Short and Long Term Effects of Breast Feeding on Child Health*, 478: 327.

Kramer, Michael S, Beverley Chalmers, Ellen D Hodnett, Zinaida Sevkovskaya, Irina Dzikovich, Stanley Shapiro, Jean-Paul Collet, Irina Vanilovich, Irina Mezen, Thierry Ducruet, et al. 2001. "Promotion of Breastfeeding Intervention Trial (PROBIT): a randomized trial in the Republic of Belarus." *Jama*, 285(4): 413–420.

Kramer, Michael S, Eric Fombonne, Sergei Igumnov, Irina Vanilovich, Lidia Matush, Elena Mironova, Natalia Bogdanovich, Richard E Tremblay, Beverley Chalmers, Xun Zhang, et al. 2008*a*. "Effects of prolonged and exclusive breastfeeding on child behavior and maternal adjustment: evidence from a large, randomized trial." *Pediatrics*, 121(3): e435–e440.

Kramer, Michael S, Frances Aboud, Elena Mironova, Irina Vanilovich, Robert W Platt, Lidia Matush, Sergei Igumnov, Eric Fombonne, Natalia Bogdanovich, Thierry Ducruet, et al. 2008*b*. "Breastfeeding and child cognitive development: new evidence from a large randomized trial." *Archives of general psychiatry*, 65(5): 578–584.

Kramer, Michael S, Lidia Matush, Irina Vanilovich, Robert W Platt, Natalia Bogdanovich, Zinaida Sevkovskaya, Irina Dzikovich, Gyorgy Shishko, Jean-Paul Collet,

Richard M Martin, et al. 2007. "Effects of prolonged and exclusive breastfeeding on child height, weight, adiposity, and blood pressure at age 6.5 y: evidence from a large randomized trial." *The American journal of clinical nutrition*, 86(6): 1717–1721.

Kramer, Michael S, Richard M Martin, Jonathan A C Sterne, Stanley Shapiro, Mourad Dahhou, and Robert W Platt. 2009. "The double jeopardy of clustered measurement and cluster randomisation." *BMJ*, 339.

Kramer, Michael S, Tong Guo, Robert W Platt, Stanley Shapiro, Jean-Paul Collet, Beverley Chalmers, Ellen Hodnett, Zinaida Sevkovskaya, Irina Dzikovich, Irina Vanilovich, et al. 2002. "Breastfeeding and infant growth: biology or bias?" *Pediatrics*, 110(2): 343–347.

Krol, Kathleen M, and Tobias Grossmann. 2018. "Psychological effects of breastfeeding on children and mothers." *Bundesgesundheitsblatt-Gesundheitsforschung-Gesundheitsschutz*, 61(8): 977–985.

Lien, EL, C Richard, and DR Hoffman. 2018. "DHA and ARA addition to infant formula: Current status and future research directions." *Prostaglandins, Leukotrienes and Essential Fatty Acids*, 128: 26–40.

Lupton, Joanne R, JA Brooks, NF Butte, B Caballero, JP Flatt, SK Fried, et al. 2002. "Dietary reference intakes for energy, carbohydrate, fiber, fat, fatty acids, cholesterol, protein, and amino acids." *National Academy Press: Washington, DC, USA*, 5: 589–768.

Martin, Richard M, Michael S Kramer, Rita Patel, Sheryl L Rifas-Shiman, Jennifer Thompson, Seungmi Yang, Konstantin Vilchuck, Natalia Bogdanovich, Mikhail Hameza, Kate Tilling, et al. 2017. "Effects of promoting long-term, exclusive breastfeeding on adolescent adiposity, blood pressure, and growth trajectories: a secondary analysis of a randomized clinical trial." *JAMA pediatrics*, 171(7): e170698–e170698.

Martin, Richard M, Rita Patel, Michael S Kramer, Lauren Guthrie, Konstantin Vilchuck, Natalia Bogdanovich, Natalia Sergeichick, Nina Gusina, Ying Foo, Tom Palmer, et al. 2013. "Effects of promoting longer-term and exclusive breastfeeding on adiposity and insulin-like growth factor-I at age 11.5 years: a randomized trial." *Jama*, 309(10): 1005–1013.

Oken, Emily, Rita Patel, Lauren B Guthrie, Konstantin Vilchuck, Natalia Bogdanovich, Natalia Sergeichick, Tom M Palmer, Michael S Kramer, and Richard M Martin. 2013. "Effects of an intervention to promote breastfeeding on maternal adi-

posity and blood pressure at 11.5 y postpartum: results from the Promotion of Breastfeeding Intervention Trial, a cluster-randomized controlled trial." *The American journal of clinical nutrition*, 98(4): 1048–1056.

Organization, World Health, et al. 2006. "WHO child growth standards: length/height-for-age, weight-for-age, weight-for-length, weight-for-height and body mass index-for-age: methods and development."

Owen, Christopher G, Emily Oken, Alicja R Rudnicka, Rita Patel, Jennifer Thompson, Sheryl L Rifas-Shiman, Konstatin Vilchuck, Natalia Bogdanovich, Mikhail Hameza, Michael S Kramer, et al. 2018. "The Effect of Longer-Term and Exclusive Breastfeeding Promotion on Visual Outcome in Adolescence." *Investigative ophthalmology & visual science*, 59(7): 2670–2678.

Patel, Rita, Emily Oken, Natalia Bogdanovich, Lidia Matush, Zinaida Sevkovskaya, Beverley Chalmers, Ellen D Hodnett, Konstantin Vilchuck, Michael S Kramer, and Richard M Martin. 2013. "Cohort profile: the promotion of breastfeeding intervention trial (PROBIT)." *International journal of epidemiology*, 43(3): 679–690.

Skugarevsky, Oleg, Kaitlin H Wade, Rebecca C Richmond, Richard M Martin, Kate Tilling, Rita Patel, Konstantin Vilchuck, Natalia Bogdanovich, Natalia Sergeichick, George Davey Smith, et al. 2014. "Effects of promoting longer-term and exclusive breastfeeding on childhood eating attitudes: a cluster-randomized trial." *International journal of epidemiology*, 43(4): 1263–1271.

Vidmar, Suzanna, John Carlin, Kylie Hesketh, and Tim Cole. 2004. "Standardizing anthropometric measures in children and adolescents with new functions for egen." *The Stata Journal*, 4(1): 50–55.

WHO, World Health Organization. 2020. "The WHO Child Growth Standards." URL: https://www.who.int/childgrowth/standards/en/, Accessed: 2020-07-16.

Yang, Seungmi, Richard M Martin, Emily Oken, Mikhail Hameza, Glen Doniger, Shimon Amit, Rita Patel, Jennifer Thompson, Sheryl L Rifas-Shiman, Konstantin Vilchuck, et al. 2018. "Breastfeeding during infancy and neurocognitive function in adolescence: 16-year follow-up of the PROBIT cluster-randomized trial." *PLoS medicine*, 15(4): e1002554.
